# Reproducible Human Neural Circuits Printed with Single-Cell Precision Reveal the Functional Roles of Ephaptic Coupling

**DOI:** 10.1021/acsnano.5c11482

**Published:** 2025-10-26

**Authors:** Johannes Striebel, Rouhollah Habibey, Daniel Wendland, Helge Gehring, Elizaveta Podoliak, Julia S. Pawlick, Kritika Sharma, Alex H. M. Ng, Wolfram Pernice, Volker Busskamp

**Affiliations:** † Faculty of Medicine, Department of Ophthalmology, 9374 University of Bonn, Ernst-Abbe-Str. 2 53127 Bonn, Germany; ‡ University Hospital Bonn, Venusberg-Campus 1 53127 Bonn, Germany; § Institute of Physics and Center for Nanotechnology, 9185 University of Münster, Heisenbergstraße 11 48149 Münster, Germany; ∥ Kirchhoff-Institute for Physics, University of Heidelberg, Im Neuenheimer Feld 227 69120 Heidelberg, Germany; ⊥ Department of Genetics, Blavatnik Institute, 630058 Harvard Medical School, 77 Avenue Louis Pasteur, Boston, Massachusetts 02115, United States; # Wyss Institute for Biologically Inspired Engineering at Harvard University, 201 Brookline Avenue, Boston, Massachusetts 02215, United States

**Keywords:** in vitro stem cell-derived neuronal networks, reproducible neuronal network formation, ephaptic coupling, direct laser writing, microscaffolds, microelectrode array, single-cell resolution

## Abstract

Although in vitro neuronal models are accessible and versatile systems for functional electrophysiological studies, the spontaneous and random formation of neural circuits often compromises the structural control and reproducibility. Here, we introduce a robust method for engineering human neuronal networks in vitro with single-cell precision and reproducibility. Our integrated platform combines direct laser-written microstructure templates and soft lithography-based fabrication of microscaffolds with functional multielectrode array recordings. This system enables high-throughput production of diverse circuit designs and allows for the exact placement of neurons within confined microenvironments. The system enables precise recording of spontaneous neuronal activity, as well as electrical and optogenetic stimulations. Using this approach, we constructed reproducible, bottom-up neuronal circuits composed of a defined number of human neurons. As a proof of principle, we employed these circuits to investigate ephaptic coupling, which refers to the modulation of neuronal activity by endogenous electric fields. Although it is believed to play a role in neural computations and cardiac conduction and is associated with epilepsy and arrhythmia, its mechanisms are unclear due to limitations in experimental models, both in vivo and in vitro. By controlling axonal proximity within microchannels and the number of neurons in the engineered circuits, we can quantify ephaptic coupling at different strengths, which validates theoretical predictions, including reduced action potential velocity, increased activity synchronization, and lower stimulation thresholds. Furthermore, the platform has broad potential for studying synaptic and nonsynaptic interactions, myelination processes, advancing disease modeling, and fundamental neuroscience research.

## Graphical abstract

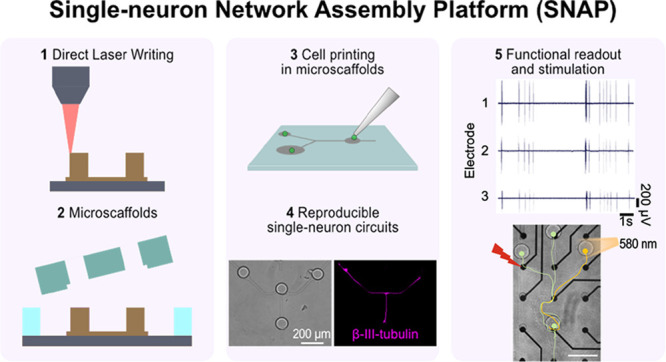

To investigate neuronal processes and network function, both in vivo and in vitro approaches are important. While in vivo experiments allow us to observe processes during natural behavior, it is more difficult to get full control over and readout from the networks due to limitations of readout methods and inaccessibility. In vitro networks have the advantage of easier access, making it possible to perform precise interventions and have a large-scale electrophysiological readout of network dynamics.^1,2^ The emergence of human-induced pluripotent stem cells (hiPSCs) and methods to derive various cell types like neurons from them was an important step for the development of in vitro systems like organ-on-chip or more specifically brain-on-chip technologies. These methods became useful tools for fundamental neuroscience research^3^ as well as for disease modeling and drug development. For biomedical applications, human cells are more relevant in clinical contexts. They help bridge the translational gap and provide a scalable cell source with fewer ethical concerns than some alternative models.

So far, in vitro networks have suffered from several drawbacks. First, most experiments have a large intersample variability in network architecture since network formation is mostly random. This introduces additional degrees of freedom in the system, making the results less comparable. This can be partially addressed by using mechanical confinements with predefined structures such as microfluidics or by surface patterning with chemical cues.^4–8^ Second, uncontrolled growth and network structure contribute to the complexity of data acquisition and analysis. Third, there is variation in network morphology over time, making longitudinal studies more difficult.^9^

These points are an issue when the fundamental building blocks of neuronal systems are to be studied. The effects of synapses, specific structural network motifs, or small network perturbations are easily masked by network effects and a lower signal-to-noise ratio to which large and randomly organized networks contribute to. Network motifs are small connectivity patterns of few neurons which are hypothesized to be the functional building blocks of neuronal networks.^10,11^ Isolated study of such network motifs was so far done in ex vivo preparations,^12^ but building such motifs reproducibly has not been reported so far.

To study such basic effects, a bottom-up approach to neuroscience with a tighter control of the circuit structure and reproducibility down to the single-cell level is needed and has long been investigated. It has been a challenge to precisely place neurons, make them connect in a predefined way, and keep them alive over extended periods of time.^13^ Different methods have been used to create such networks, including surface functionalization and microscaffolds for guidance of network development.^14–18^ Most approaches have so far relied on a random seeding approach, with cells falling into the intended locations like through a sieve.^19^ Although such an approach allows one to fabricate many circuits in parallel in a quick way, it comes to its limits when different cells or neuronal subtypes need to be placed within neuronal circuits or many defined circuits with a larger number of neurons in a circuit are to be created reproducibly. Precise placement of cells was previously demonstrated with fluidic force microscopy.^20,21^ One of the main obstacles was the survival of single neurons after placement to get functional and complete circuits.^13^

Here, we report the development of the s**ingle-neuron network assembly platform (SNAP)**, a method to construct neuronal circuits from bottom up with single-cell resolution in a reproducible manner. To our knowledge, this is the first approach capable of reliably recreating defined neural circuits with such precision and reproducibility. SNAP integrates direct laser writing (DLW) for template fabrication, rapid soft lithography-based replication, and a straightforward, cost-effective single-cell printing technique to place neurons into microchannel devices aligned on multielectrode arrays (MEAs). MEAs allow large-scale, high-throughput extracellular functional recordings over long time periods as opposed to patch clamping. The resulting circuits have a well-defined architecture and stable function, overcoming variability typical of in vitro models. Furthermore, SNAP supports both spontaneous and controlled network activities through electrical and optogenetic stimulations, providing a powerful platform for probing neuronal dynamics, as well as inclusion of several cell types in a circuit. MEA technology interfaces well with this platform and enables longitudinal measurements over extended periods of time.

We leveraged these capabilities to experimentally investigate ephaptic coupling, a fundamental biophysical phenomenon, in human neuronal networks in vitro under controlled, tunable, and reproducible conditions. Ephaptic coupling is a mechanism of neuronal interaction, in addition to chemical and electrical synapses. Electric fields present in the neuronal tissue due to the flow of ions and charged neuronal compartments have been shown to affect functional properties such as signal propagation or synchronization in the surrounding tissue. For example, propagation speed and synchronization were reported in crab nerves.^22^ Because ephaptic coupling is mediated by electric fields, it is not easily manipulated experimentally and is, therefore, notoriously difficult to study in vivo and in vitro. While computational studies have extensively explored the potential consequences of ephaptic coupling,^23–31^ there were some experimental studies that have had to use external fields or advanced methods to study ephaptic effects.^32–35^ However, its role in neural computation and its functional significance in the brain are still open questions in need of further experimental validation. There is evidence that ephaptic coupling affects olfaction,^36^ the retina,^37,38^ and possibly higher-level computations of the brain.^28,32,39^ Cardiac conduction may also be supported by ephaptic mechanisms in addition to gap junctions.^40^ It is suggested that ephaptic coupling may also contribute to pathological conditions such as epileptic seizures^26,27,41–43^ and arrhythmias.^24,25,44,45^ However, further research is needed to fully elucidate the functional significance and impact of ephaptic coupling across these physiological and pathological contexts. Using SNAPs, we could precisely measure the effects of ephaptic coupling on electrophysiological network dynamics. For example, we measured a decreased action potential (AP) velocity and increased synchronicity with an increasing number of participating neurons in a circuit without the need for complex experimental setups. These results validate the predictions of computational models and exemplify the ability of this platform for the theoretical hypothesis-led design of neuroscientific experiments.

## Results and Discussion

### Preparation of Microstructures and Neuronal Circuits

To build SNAPs, we chose to use microfluidics as a scaffold, in which human neurons can grow and be guided to connect in a predefined manner. This is a well-established method for engineering neural circuits^17^ and has several advantages over other methods. First, microfluidics interface well with MEAs, which facilitates electrophysiological readout of neuronal activity over the lifetime of the circuit. Second, developing neural networks in vitro show substantial changes in morphology over time.^9^ Microfluidics keeps the circuit structure constant over time and the neurites close to the readout electrodes. Third, the stable microenvironment within microfluidic devices can shield cells from mechanical perturbations, increasing the survival of neuronal circuits during media changes or recording sessions. Alternative approaches like microcontact printing of adhesive molecules showed unspecific growth and unwanted connections as well as morphological instability over time.^16^

We developed a method for fast large-scale production of microfluidic scaffolds in PDMS from direct laser-written masters. DLW offers great flexibility in circuit designs and has no need for mask fabrication, which makes it ideal for small batch custom-design manufacturing as opposed to classical photolithography. After direct laser writing our custom designs, they were subjected to a multistep soft lithography process (Figure 1a) to get many stencils for microscaffold fabrication. First, the DLW-masters were replicated in epoxy resin. From these replicas, many PDMS molds were prepared that were then used to create stencils made of epoxy on glass substrates. A height-matched frame around the structures allows fabrication of microscaffolds with through-holes that serve as microwells by a PDMS molding step in 30 min.

**Figure 1:**
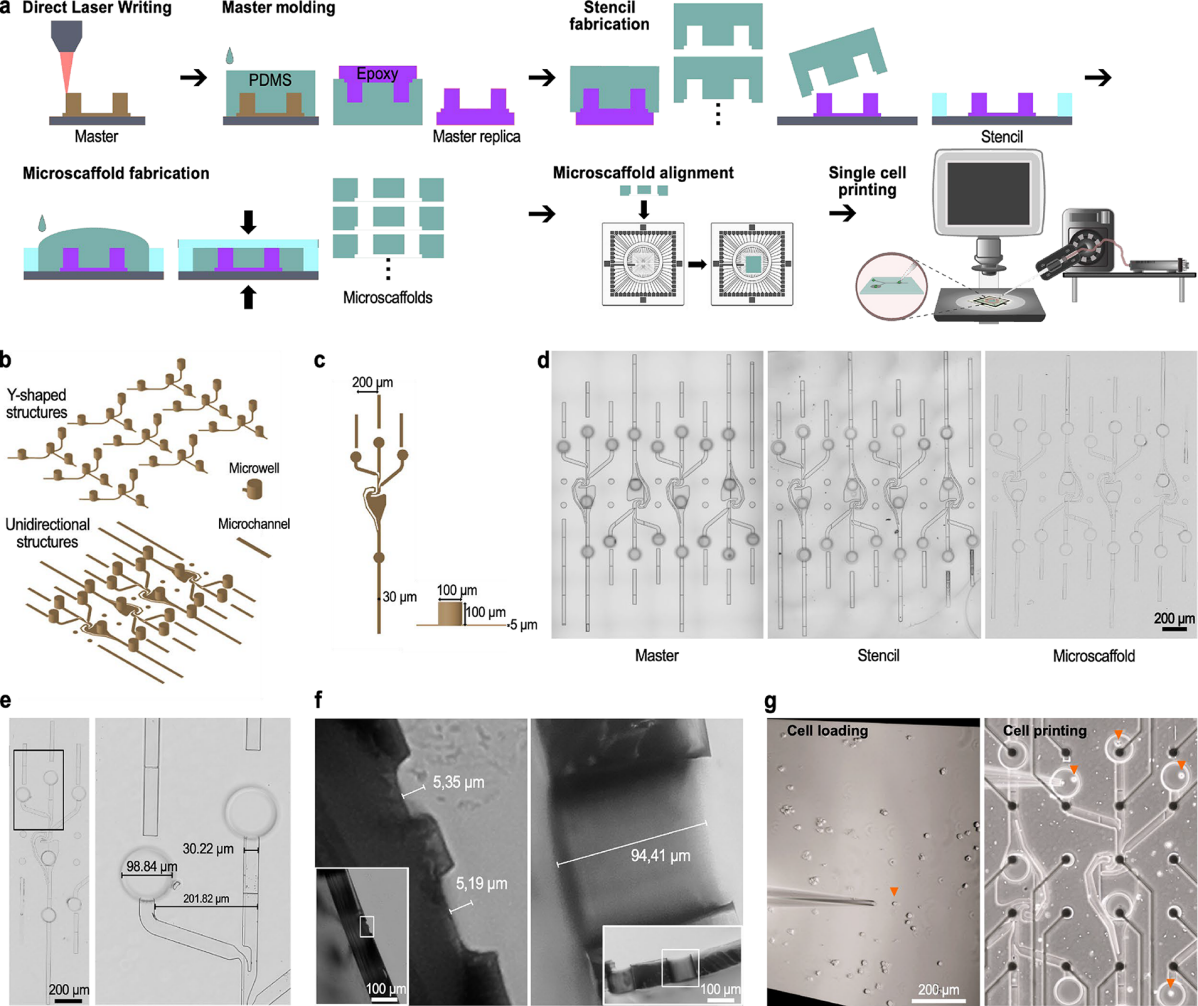
SNAP for the parallel construction of reproducible neuronal circuits at single-cell resolution by printing neurons in microscaffolds. (a) Microscaffold designs for neuronal circuit construction are prepared via direct laser writing. After they were replicated in epoxy via soft lithography, many stencils consisting of epoxy on a glass substrate are prepared for parallel manufacturing of microscaffolds. PDMS microscaffolds are then aligned on MEA electrodes.^54^ Single neurons are placed in microwells with a microinjector attached to a micromanipulator. (b) Two different designs (Y-shaped and unidirectional structures) were written in an array to fit several structures on one MEA. (c) Dimensions of microwells and microchannels used in the designs. (d) Microscopy images of a representative master, stencil, and microscaffold. (e,f) Size measurements of a representative microscaffold confirm the accurate reproduction of the designs. (g) Left, single cell (orange arrow) is being taken up by the micropipette. Right, cell release from the micropipette into the microwell (Movie S1). All single cells placed in microwells are marked with an orange arrow. Microfluidic structure is aligned to the recording electrodes (black dots) of a MEA.

Two different microscaffold designs were used in this study: a Y-shaped structure for three-neuron circuits and a design for unidirectional circuit formation of four neurons (Figure 1b,c). Each device aligned with one MEA incorporated four Y-shaped motifs (Figure 1d). The dimensions of the designs were chosen to match the electrode layout of standard MEAs.^46^ A microchannel height of 5 μm was chosen to avoid neuron somas to migrate into the microchannels^47^ keeping them at their intended position, the microwells (Figure 1f). Accurate manufacturing and replication of the designs throughout the fabrication process and down to the microscaffolds are possible (Figure 1d–f). For improved readout, electrodes were positioned within the microchannels (Figure 1g).^46^

We used the transgenic human inducible neurogenin (iNGN) cell line in which the overexpression of the transcription factors neurogenin-1 and -2 drives hiPSCs into postmitotic neurons in just 4 days.^48^ Simply pipetting neurons onto the microfluidic scaffolds did not ensure that each microwell contained exactly one cell. To build the circuits in a tightly controlled, bottom-up fashion and to incorporate multiple cell types in a reproducible manner, we were in need of a method for precise placement of individual cells in each microwell (Figure 1a,g). We were inspired by blastocyst injection, a technique used to generate transgenic mouse lines by transferring stem cells with a micropipette.^49,50^ We developed a custom setup that included a microscope with a micromanipulator holding a glass pipet connected to a microinjector (Figure S1b, Movie S1). A critical aspect of the setup was to match the tip diameter of the micropipet to the cell dimensions to ensure efficient and reliable cell transfer. Taking up of single cells and placing them in a microwell took a few tens of seconds. Consequently, an array of microscaffolds containing 16 neurons (Figure 1c,d) could be seeded in approximately 20 min, including all steps like adding cell suspension and washing as detailed in the methods section.

The next challenges were biocompatibility and neuronal survival as single cells per well. In stem cells, the ROCK pathway is commonly inhibited after passaging to avoid anoikis.^51^ Recently, a more potent four-substance cocktail (CEPT) has been reported for this purpose, building on the idea of ROCK pathway inhibition.^52^ We hypothesized that these supplements might help the initial survival of cells, until a circuit is established. Indeed, the addition of a ROCK pathway inhibitor (ROCKi) improved survival up to 5 days after seeding but showed no significant improvement thereafter (Figure S1c). In contrast, the addition of CEPT dramatically improved the cell survival. Since iNGN neurons typically show robust electrophysiological activity by 14 days post induction (dpi), with functional synapses present by 21 dpi,^53^ we had a method that allowed us to construct identical circuits and record functional activity from them in a manageable batch size.

We also created SNAPs composed of two different neuronal cell types, iNGN and EMX1 cells (Figure S1d). This exemplifies that our method can be used to construct more complex circuits that include more than one cell type in a reproducible manner. Different neuronal subtypes such as excitatory and inhibitory neurons, myelinating cells such as oligodendrocytes, or glial cells could be combined.

### SNAPs are Sensitive to Synaptic Intervention

First, we fabricated circuits consisting of three neurons in a Y-shaped microfluidic structure (Figures 1b and 2a). Immunocytochemistry confirmed the structural integrity and neuronal identity of the cells (Figure 2a). EMX1 and iNGN neurons show a similar survival rate over time (Figures 2b, S1f and S2d). Out of 40 circuits, 13 complete circuits were available for experiments after 22 dpi (Figure 2c). To verify that the SNAPs were functionally connected via excitatory synapses, we added a mixture of the AMPA receptor antagonist (NBQX) and the NMDA receptor antagonist (AP5) to the cultures. Blocking excitatory transmission should lead to a change in firing patterns, indicating functional synaptic connections. We observed significant changes in the baseline activity with the antagonists added to the SNAPs for both iNGN and EMX1 neurons. As a control, we used cultures in which populations of neurons were added to the microwells instead of single neurons (Figure S1e). There was also a change in the raw activity traces in the controls, although not as pronounced (Figure 2d). Interspike interval (ISI) histograms are fundamental measures of electrophysiological activity. ISI is commonly used to characterize firing regularity, burstiness, and excitability. These are hallmarks of excitatory synaptic connections that can produce synchronized or bursting activity within a network. For SNAPs, the ISI histograms were significantly changed after the addition of the antagonists, while this change was subtle for the population controls (Figure 2e). Using the Kullback–Leibler (KL) divergence,^55^ we were able to quantify how much the two histograms (baseline vs NBQX+AP5) differ. Both iNGNs and EMX1 showed a significant increase in KL divergence for SNAPs compared to population controls (Figure 2f,g), indicating that SNAPs provide a more sensitive readout to interventions than populations of cells. This could be due to the dense activity in neuronal populations masking such effects.

**Figure 2:**
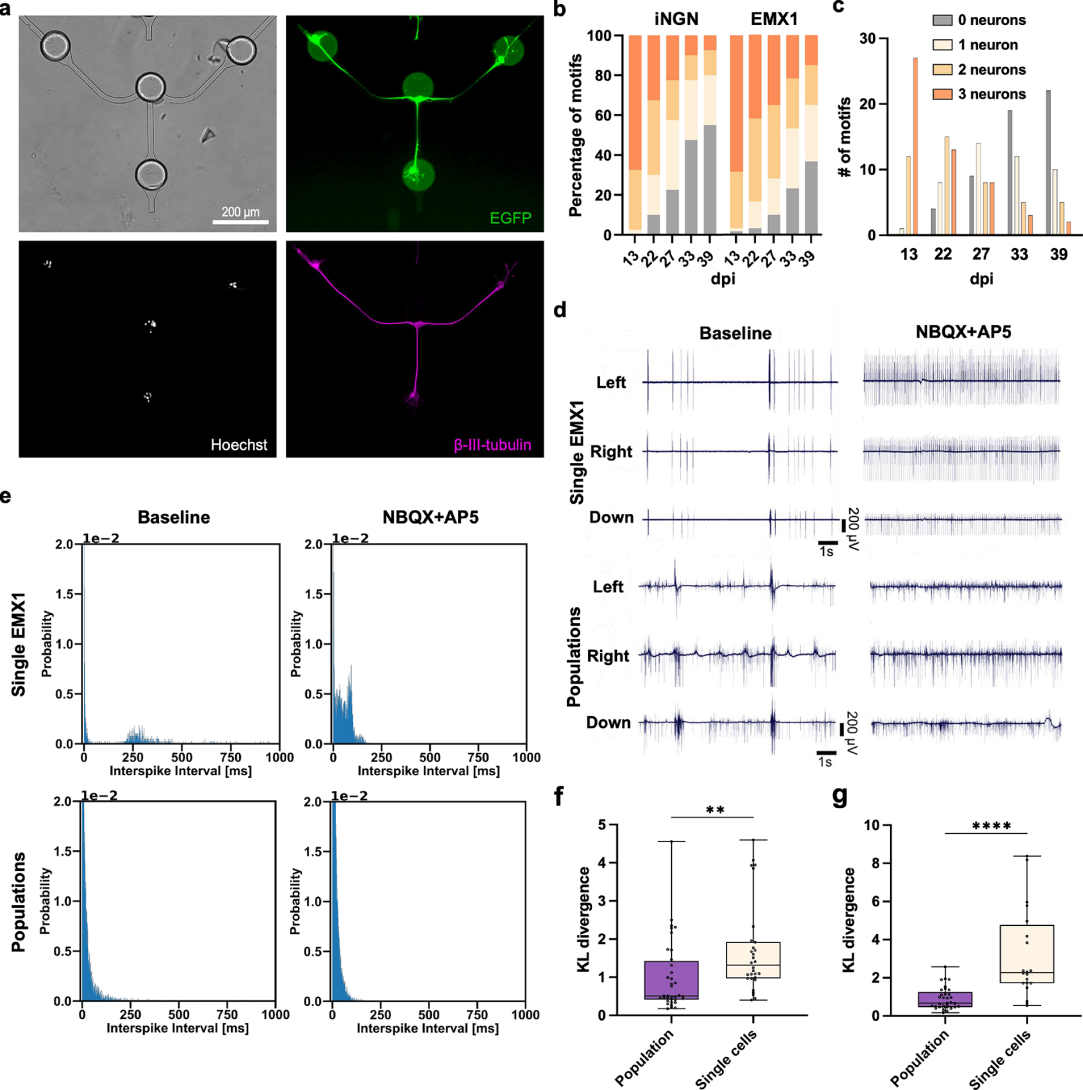
SNAPs are sensitive to synaptic antagonists. (a) Representative neuronal circuit motif consisting of three iNGN neurons in microfluidics. Circular structures in the bright-field image are microwells with single neurons placed in outer wells. Immunofluorescence images of β-III-tubulin, Hoechst, and constitutively expressed EGFP. (b) Percentage of motifs containing the respective number of iNGN and EMX1 neurons in three-neuron circuit motifs over time at different dpi (iNGN: *n* = 64 circuits; EMX1: *n* = 60 circuits). (c) Number of circuit motifs with the respective number of iNGN neurons per dpi. Ten samples with four motifs each were monitored. (d) Representative raw traces of three electrodes (left, right, down channels) in one circuit motif of a SNAP (3× EMX1 neurons) and populations of EMX1 neurons seeded in the same structure. Left, baseline recordings. Right, with excitatory blockers NBQX+AP5. (e) ISI histograms of the activity measured in representative electrodes of a circuit motif with single and population EMX1 neurons. Left, baseline recordings. Right, after adding excitatory blockers NBQX+AP5. For the single-cell case, a change in the histogram shape is visible. (f,g) KL divergence of baseline vs NBQX+AP5 ISI histograms for single neuron and population circuits. Both EMX1 (f) and iNGN (g) neurons showed a statistically significant increase in KL divergence for the single neuron circuits compared to populations, indicating a higher sensitivity to interventions via synaptic antagonists. In both plots, box denotes 25th to 75th percentile with median; whiskers denote min to max. Two-tailed Mann–Whitney test (EMX1: *n* = 36 and 30 electrodes for population and single cell, respectively; iNGN: *n* = 33 and 20 electrodes for population and single cell, respectively); ** *P* ≤ 0.01, **** *P* ≤ 0.0001.

### Ephaptic Coupling in SNAPs

For ephaptic coupling to be effective, it is important to bring the axons into close contact. Therefore, we created specific microscaffolds that unidirectionally guide axons of four neurons together (Figure 3a). We were inspired by a previously published stomach-shaped design that showed unidirectional guidance of axons.^15^ First, the reservoir geometry promotes axonal growth in the desired direction. Second, the microchannel dimensions and configurations prevent reverse growth. Additionally, a small rebound microchannel connected to the side of the stomach-shaped reservoir redirects axons growing backward, guiding them back into the forward path. We further included narrowing and sharp turns in the channels where three microchannels converge into the stomach module, thereby preventing the axon from extending into neighboring modules.

**Figure 3:**
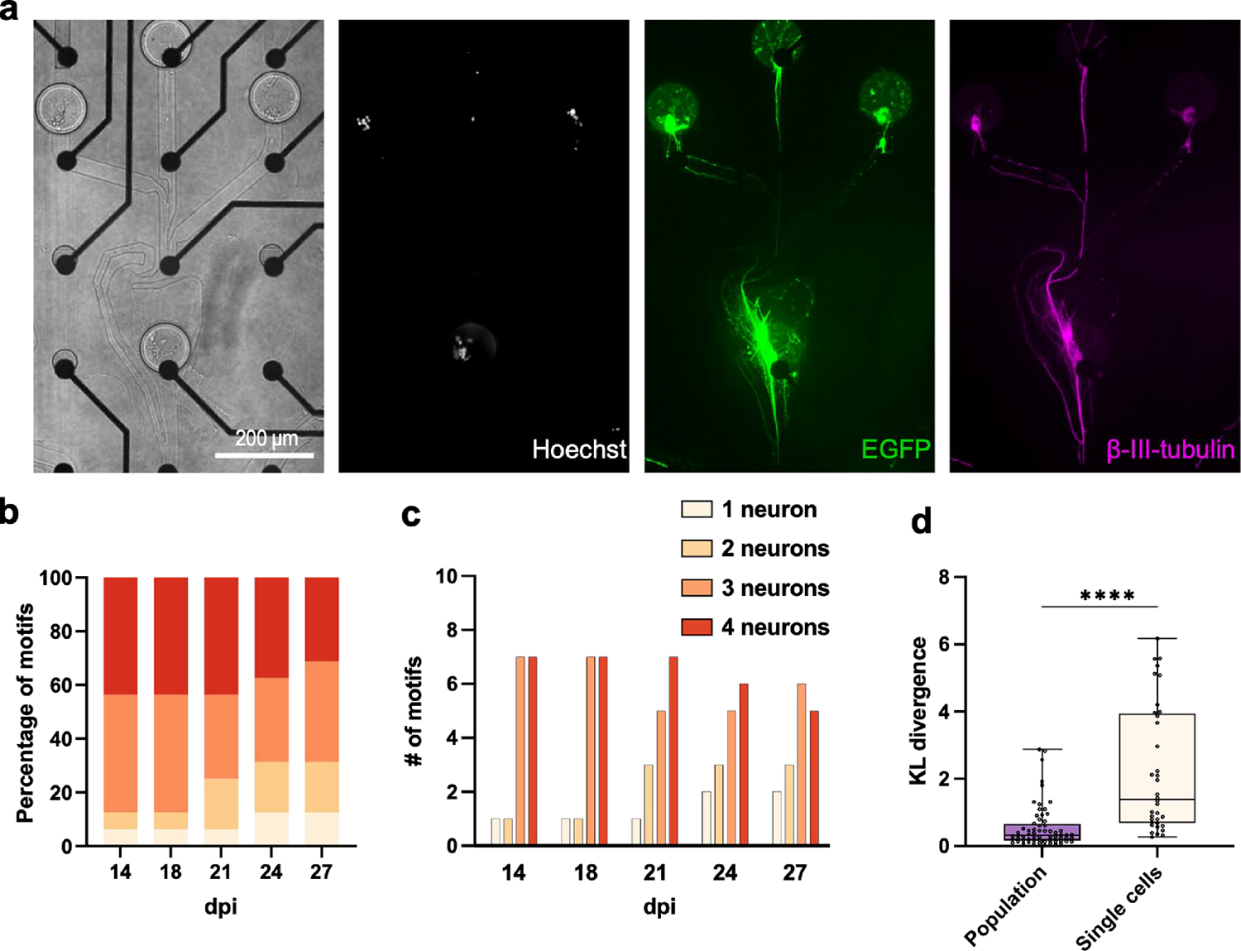
Directional neuronal circuits with single neuron resolution. (a) Representative neuronal circuit motif consisting of four iNGN neurons in microscsaffolds. Circular structures in the bright-field image are microwells with single cells placed in all wells. The stomach-shaped structure in the middle guides neurites downward.^13,15^ Immunofluorescence images of β-III-tubulin, Hoechst, and constitutively expressed EGFP. (b) Percentage of motifs containing the respective number of viable cells at several days dpi for directional four-neuron circuit motifs (iNGN: *n* = 16 circuits). (c) Number of circuit motifs containing the respective number of iNGN neurons per dpi. Four samples with four motifs each were monitored. (d) KL divergence of baseline vs NBQX+AP5 ISI histograms for directional SNAPs and population control. Single-cell directional circuits show a statistically significant increase in KL divergence compared to populations of iNGNs in the same microfluidic structure, confirming functional excitatory synapses and indicating a higher sensitivity to interventions via synaptic antagonists. Box plots: 25th to 75th percentile with a median. Whiskers: min to max. Two-tailed Mann–Whitney test (*n* = 64 and 36 electrodes for population and single cell, respectively). **** *P* ≤ 0.0001.

One MEA with 60 electrodes in an 8 × 8 array accommodated a microfluidic device with four identical structures is shown in Figure 1b. Notably, the survival of neurons in these axon-guided structures was increased compared with the Y-shaped structure (Figures 3b,c and S2a–c). Our results suggested that the microchannel geometry can influence cell viability, potentially by providing a more favorable microenvironment. Additionally, the enforced close contact between neurites within the confined space may promote the formation of synaptic contacts. We also tested that the circuits were responsive to excitatory synaptic antagonists (NBQX+AP5), which allows blocking of transmission other than ephaptic coupling (Figure 3d). Similar changes in electrophysiological activity and ISI histograms could be observed as quantified by the KL divergence. Cross-correlation analysis of the recorded activity confirmed directional signal propagation within the microfluidic structures, supporting the functionality of the device in guiding axons along the same direction (Figure S2f).

Next, we focused on ephaptic coupling characteristics, such as changes in the AP velocity. Within SNAPs, we were able to precisely measure AP velocity changes with correlation to the number of neurons in a circuit. We observed a significant decrease in the AP velocity as more neurons interacted (Figure 4a). To confirm that this effect was not mediated by synaptic delays, we measured the AP velocity under excitatory synaptic antagonists NBQX+AP5 and electrical synaptic antagonist carbenoxolone (CBX), which did not significantly change the result (Figure 4b). Our microfluidic structures have regions where axons are separated and maintained individually and are later brought into contact to bundle. If ephaptic coupling is involved, we would have expected the AP velocity to be different in the areas of individual axons compared to the bundled areas. Indeed, we observed a significant decrease in the AP velocity between these regions (Figure 4c). Our experimental setup facilitated a detailed study of individual circuits longitudinally over time. Figure 4d shows the evolution of AP velocity for different dpi values in two circuits. Some neurons degenerate over time, gradually lowering the strength of ephaptic coupling when changing from bundled axons to individual axons. This resulted in a significant increase in the AP velocity, further indicating that the change in the cell number affected the propagation of APs. We observed a direct influence of axonal interaction length on the AP velocity as predicted by theory (Figure 4e).^23^ Immediately after seeding, we observed that axons were mostly separate, but over time, they began to bundle together, resulting in a decrease in the AP velocity. This suggests that the increased interaction length resulting from axon bundling influenced the AP propagation. After the degeneration of two of the four neurons in the same circuit, the AP velocity increased again, suggesting that the reduction in the number of neurons may have restored more efficient propagation. This change highlights the complex relationship between axonal organization and the dynamics of AP conduction in the circuit.

**Figure 4:**
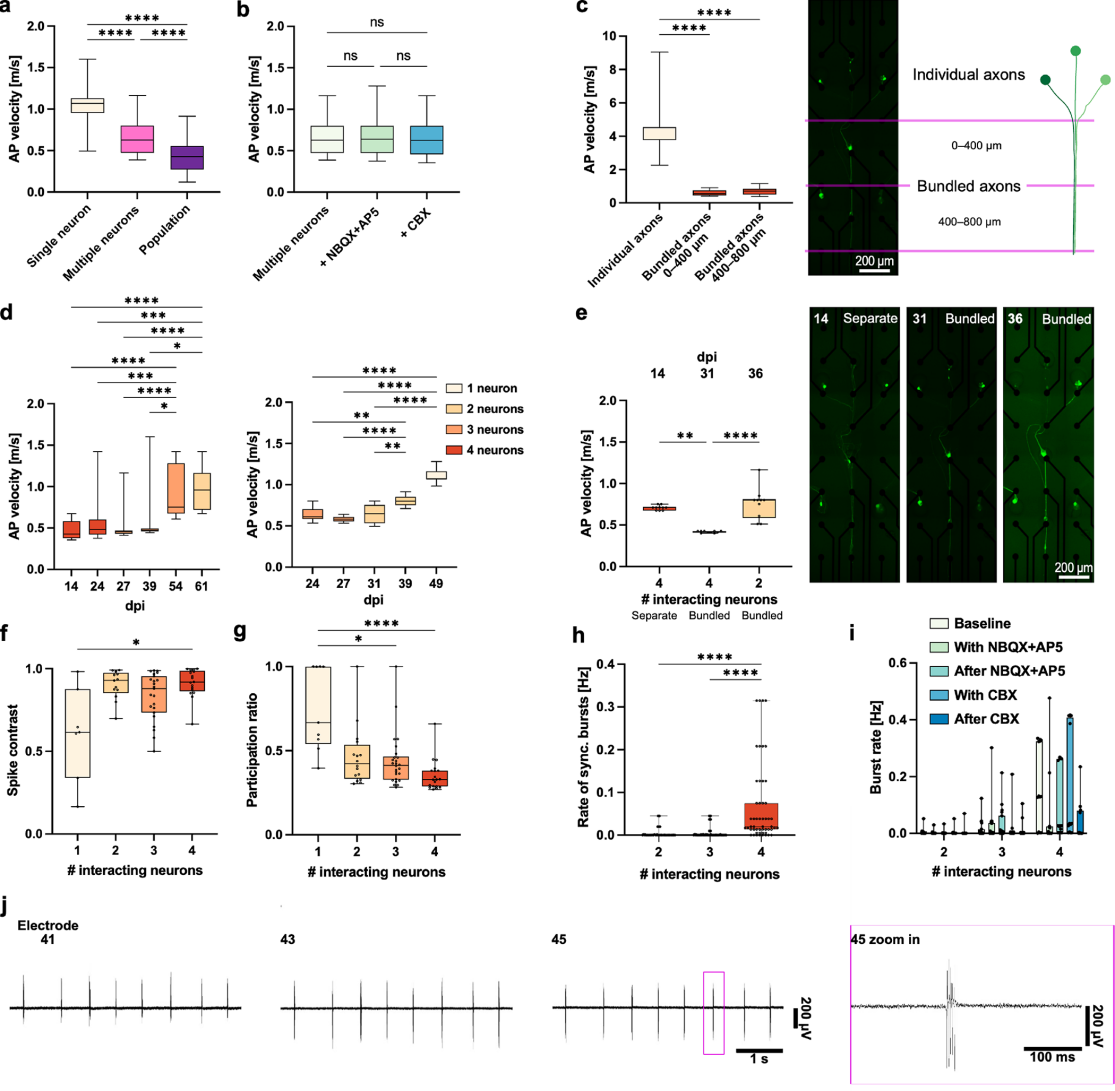
Ephaptic coupling in directional single neuron circuits. (a) Velocity of AP propagation for single iNGN neurons, multiple (2–4) neurons, and neuronal populations in directional microfluidic structures. AP velocity decreases significantly with the number of neurons. Kruskal–Wallis test with Dunn’s multiple comparisons test (single neurons: *n* = 60 APs; multiple neurons: *n* = 100 APs; population: *n* = 79 APs). (b) AP velocity does not differ significantly for multiple neurons after adding excitatory blockers NBQX+AP5 or gap junction blocker CBX. Two-tailed Mann–Whitney test (multiple neurons: *n* = 100 APs; multiple neurons + NBQX+AP5: *n* = 100 APs; multiple neurons + CBX: *n* = 100 APs). (c) AP velocity differs significantly in areas of individual vs bundled axons. Kruskal–Wallis test with Dunn’s multiple comparisons test (individual axons: *n* = 29 APs; bundled axons 0–400 μm: *n* = 50 APs; bundled axons 400–800 μm: *n* = 50 APs). (d) Development of AP velocities over time for two-directional circuit motifs. Velocity increases significantly in the area of bundled axons with the number of cells reducing in the circuit by natural cell death. Kruskal–Wallis test with Dunn’s multiple comparisons test (left: *n* = 20, 20, 20, 19, 19, and 20 APs at dpi 14–61; right: *n* = 20, 20, 22, 20, and 19 APs at dpi 24–49). (e) Change of AP velocity in a circuit motif over time. At 14 dpi, axons are mostly separate leading to a short interaction length and higher velocity as compared to 31 dpi when axons have bundled together. After two cells in the circuit degenerated, the velocity increased again. Kruskal–Wallis test with Dunn’s multiple comparisons test (*n* = 10 APs for each condition). Bright-field images see Figure S2g. (f) Spike contrast as a measure of synchronized activity in the circuits increases significantly with the number of interacting neurons in directional circuits. Kruskal–Wallis test with Dunn’s multiple comparisons test (*n* = 7, 13, 23, and 17 circuits for 1–4 neurons, respectively) after outlier identification with ROUT method (*Q* = 1%). (g) PR decreases with the number of neurons. A decrease in the PR indicates a lower dimensionality and thus more coordinated neuronal network activity. Kruskal–Wallis test with Dunn’s multiple comparisons test (*n* = 9, 16, 26, and 19 circuits for 1–4 neurons, respectively). (h) Rate of synchronized burst activity in the circuit increases significantly with the number of neurons. Kruskal–Wallis test with Dunn’s multiple comparisons test (*n* = 48, 79, and 52 circuits for two to four neurons, respectively). (i) Burst rates in circuits increase with the number of neurons, irrespective of the presence of excitatory (NBQX+AP5) or gap junction (CBX) blockers (*n* = 11, 19, and 12 circuits for each condition with two–four neurons, respectively, for each condition). (j) Exemplary raw traces measured in the area of bundled axons of one directional circuit. A strong synchronization of activity is visible. In all plots, ns *P* > 0.05, * *P* ≤ 0.05, ** *P* ≤ 0.01, *** *P* ≤ 0.001, **** *P* ≤ 0.0001. Box plots: 25th to 75th percentile with median. Whiskers: min to max.

Changes in the AP velocity in microchannels could potentially arise from mechanisms other than ephaptic coupling. For instance, changes in the ion concentration that are amplified by the microchannel confinement, especially during high activity phases, might contribute to a slowing of signal propagation. However, this effect is unlikely in our setup since each microchannel contains only a very limited number of axons (up to four per channel) with overall low firing rates (Figure 5b). Given that axons (assuming a 1 μm diameter) occupy only about 2% of the channel volume, the remaining space is largely filled with medium, allowing effective diffusion. Thus, the contribution of each AP to local ionic concentration changes would be minimal compared with the bulk ion concentrations of the medium. Additionally, previous work has shown a quick diffusion of ions in microfluidics resulting in fast equilibration of gradients.^56^ Another study supports the negligibility of this effect by showing that increased activity through higher number of axons or increased bursting in channels did not slow conduction.^57^ Future studies should refine microfluidic designs to more directly separate ephaptic effects from possible ionic contributions while ensuring that cell health is preserved.

**Figure 5:**
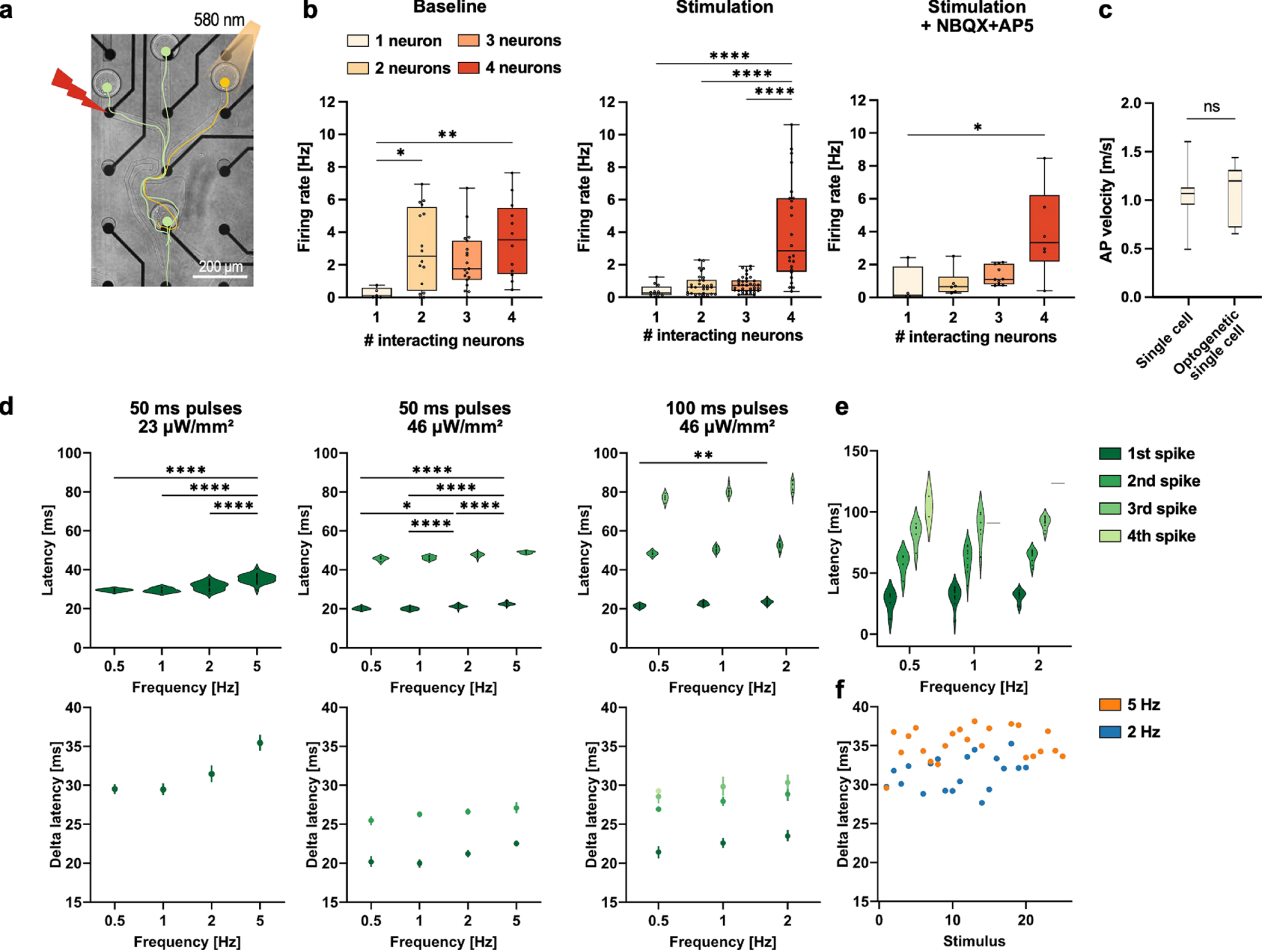
SNAPs can be stimulated electrically and optogenetically and respond reproducibly. (a) Scheme of the circuit of four neurons in a microscaffold on an MEA. Stimulation can be done electrically via defined electrodes (red) or by exposing neurons expressing optogenetic tools to light (orange). (b) Firing rates in directional SNAP circuits without stimulation (baseline) and while electrically stimulating one electrode in the circuit with and without excitatory blockers NBQX+AP5. Rates increase exponentially with the number of neurons under stimulation irrespective of the blockers being present. Kruskal–Wallis test with Dunn’s multiple comparisons test (firing rates measured in individual electrodes of 16 circuits included in baseline: *n* = 5, 16, 19, and 12; stimulation: *n* = 12, 30, 37, and 24; stimulation + NBQX+AP5: *n* = 4, 6, 10, and 6 for one to four neurons, respectively) after outlier identification with the ROUT method (*Q* = 1%). (c) AP velocity does not differ significantly for single iNGN neurons being spontaneously active and optogenetically activated APs of single iNGN neurons. Two-tailed Mann–Whitney test (Single cell: *n* = 60 APs; optogenetic single cell: *n* = 12APs). (d) Response of a single fChRimson-iNGN neuron to several trains of light stimuli. An increase in the light intensity and light pulse duration leads to more APs elicited by one light pulse. For higher stimulation frequencies, the latency between light on and AP peak increases. Left, middle: *n* = 5, 10, 20, and 25 pulses; right: *n* = 5, 10, and 10 pulses for 0.5–5 Hz, respectively. Statistical analysis for first APs at different frequencies: Shapiro-Wilk and Kolmogorov–Smirnov tests for normality passed in all cases (alpha = 0.05) followed by ordinary one-way ANOVA with Tukey’s multiple comparisons test. Bottom plots: mean ±95% confidence interval. (e) Same behavior is observed for a different fChRimson-iNGN neuron (100 ms pulses, 46 μW/mm2). Lines represent a fourth AP for 1 and 2 Hz. *N* = 5, 10, and 10 pulses for 0.5–2 Hz, respectively. (f) Response of a single fChRimson-iNGN neuron to two trains of light stimuli at 2 and 5 Hz (50 ms pulses, 23 μW/mm^2^). Latencies between light on and AP peak are constant over the 2 Hz stimulation episode. For the 5 Hz episode, the first stimulus elicits an AP with similar latency as in the 2 Hz pulses, but all subsequent APs have increased latencies. In all plots, ns *P* > 0.05, * *P* ≤ 0.05, ** *P* ≤ 0.01, and **** *P* ≤ 0.0001.

Another hallmark of ephaptic coupling is synchronization.^22,31,33^ The raw signals of directional SNAPs already showed synchronized activity (Figure 4j). Spikes occurring mostly in dense bursts of APs preceded, followed by longer periods of no spikes. In addition, spike contrast, a measure of synchrony, increased with the number of interacting neurons^58^ (Figure 4f).^58^ The participation ratio (PR),^59^ a measure of the dimensionality of network activity, also decreased with the number of interacting neurons, further indicating increased synchronicity (Figure 4g). Other classical measures of synchronicity such as burst rate and rate of synchronous network bursts also increased with the number of interacting neurons (Figure 4h,i).

### Stimulation of SNAPs

Ephaptic coupling is also predicted to lower the stimulation threshold of neurons within a circuit,^30^ meaning that electrical stimuli applied to networks with more cells should elicit stronger responses. We found that firing rates under stimulation increased superlinearly with the number of interacting neurons (Figure 5a). This was also observed when excitatory synaptic antagonists were applied.

In addition to electrical stimulation, we also used optogenetic stimulation to target single iNGN neurons expressing fChRimson-EYFP^60^ and measured their activity using noninvasive MEA technology. Light stimulation elicited spikes from the single neurons. As a further control for our ephaptic coupling experiments, we measured the AP velocity in the optogenetically activated neurons and found them to be comparable to spontaneously active neurons, with no decline in the AP propagation velocity (Figure 5b).

Next, we applied trains of light pulses that varied in duration, frequency, and intensity to single neurons expressing fChRimson (Figure 5c). We found that a low irradiance (23 μW/mm^2^) with a pulse duration of 50 ms elicited one AP. By nearly doubling the irradiance (46 μW/mm^2^) but keeping the 50 ms pulse duration, two APs were consistently elicited up to 2 Hz. By further increasing the pulse duration to 100 ms, we were able to measure three elicited APs. A stepwise increase in the number of APs can be achieved by modulating the light pulse intensity or duration. Notably, the latency, the time between light on and the peak of the AP, was about 10 ms longer for the low irradiance case compared with the high irradiance case. Delta latencies for subsequent spikes are defined as the time delay between the peaks of the previous and current AP. Delta latencies measured at high light intensities with 50 and 100 ms pulse durations were similar but tended to be longer with increasing spike number. We confirmed the observations by measuring a second iNGN neuron that showed the same behavior while also firing a fourth AP in rare occasions (Figure 5d). Another observation was that as the stimulation frequency increased, the latency also increased. By examining individual stimuli in the light train, we were able to confirm that the first light pulse does indeed elicit a response with the same latency as in the low-frequency case, but all subsequent spikes show increased latency (Figure 5e). Our observations could be explained by the light intensity used being close to the activation threshold of fChRimson leading to a slower increase in currents. Slowly depleting ion reserves in the cell would then lead to the change in latencies and number of fired APs with frequency and intensity. As shown here, circuits and single neurons made with the SNAP method can be stimulated electrically and optically. Optogenetic stimulation and simultaneous MEA recordings provide a more natural environment for such measurements since they are noninvasive, and ion concentrations are not controlled as opposed to patch clamp recordings. Hence, SNAP is a powerful method to characterize cells and optogenetic tools in a fast and precise way. Because of the noninvasive nature, the measurements can be carried out over extended time periods to monitor developmental aspects. The readout from only one cell expressing an optogene could enable direct fitting of characteristic parameters to the data. By incorporating neurons with optogenetic-expressing neurons in the same circuit, which is supported by SNAP, the study of information processing in circuits with high precision could be facilitated.

## Conclusions

We developed SNAP, a novel method for reproducible fabrication of neuronal circuits at single-cell resolution. Due to the use of direct laser-written master scaffolds and parallelizable manufacturing via soft lithography, this method is scalable and enables flexible designs. To increase throughput, the method could be extended to include automated cell printing^61,62^ and high-density CMOS MEA technology. A single-cell printing setup combines precise cell placement with microscaffolds and thus enables the inclusion of multiple cell types in the same structure.

We demonstrate that the endogenous electrical fields of a few neurons lead to signatures of ephaptic coupling. Direct effects such as changes in the AP velocity and synchronized activity can be measured longitudinally in a precise manner. Of note, our results experimentally validate underlying theories and modeling studies on the effects of ephaptic coupling, demonstrating the necessity and uniqueness of SNAP for neurobiological research. These findings further support the notion that ephaptic coupling is a fundamental process in neural systems. Printed neuronal circuits in microfluidic devices with single-cell resolution and persistent activity over weeks were key to obtaining these in vitro data. It is very likely that ephaptic coupling also plays an important role in in vivo systems, but this remains to be proven.

Our experimental platform serves as a tool to gain a more detailed understanding of the fundamental mechanisms underlying this phenomenon and allows the precise measurement of parameters due to purely endogenous fields. Future experiments should include myelinating cells, such as oligodendrocytes. This could provide insight into the influence of myelin on ephaptic coupling. Since myelin is also thought to shield the effect of ephaptic coupling, the model could allow the study of the same circuits with high or low ephaptic coupling, allowing for the first time the design of experiments in which the effect of ephaptic coupling can be modulated. Modifying the setup to include simultaneous patch clamp recordings may be of interest in future experiments.

We have studied spontaneous neuronal activity as well as applied electrical and optogenetic stimulation, demonstrating the potential of SNAPs to address other fundamental questions in neuroscience. Small-scale neuronal circuits are well suited to extract fundamental parameters of the network, enabling detailed validation of computational models and comparison to computational predictions.^19^ This is easier in the case of small circuits since they have fewer degrees of freedom, and thus fewer variables and network effects do not govern the neuronal dynamics. Incorporation of precise optogenetic activation facilitates the control of circuit activity at the single neuron and single spike level. Furthermore, by using patient-derived hiPSCs and generating diseased neurons, SNAP may serve as a sophisticated functional disease model with single-cell resolution. This could furthermore help in a more detailed investigation of physiological processes in health and disease and their mechanistic understanding. Additionally, our human SNAP circuits show great sensitivity to synaptic antagonists, which is extremely useful for drug-screening applications.

## Materials and Methods

### Direct Laser Writing of Microstructures

Microfluidic designs were created in Autodesk Inventor as a negative of the final structure. Custom designs were used except for the directionality-inducing structure in which a stomach-shape adapted from Girardin et al.^13^ was combined with other concepts like sharp turns for axon guidance.^63^ Microwells consisted of round structures with a diameter and height of 100 μm. In general, microchannel designs are fabricated with a thickness of around 100 μm to couple to MEAs for three main reasons: First, high aspect ratios in the templates increase the risk of damage during PDMS molding. Second, thicker devices are more difficult to attach securely to the MEA substrate, whereas thinner and more flexible devices adhere more firmly, even without plasma treatment. Third, shallow microwells enable easier and more precise seeding of neurons with micropipettes. Microchannels were 5 μm high, keeping somas from migrating into channels,^47^ and had a width of 20 μm (Y-shape) or 30 μm (directional structures). We used direct laser writing for the fabrication of the structures. The microfluidic designs were written on silicon substrates with four inch diameters to quickly create many structures. Small, 25 × 25 mm^2^ fused silica samples were used to make and reproduce single structures. First, the substrates were cleaned, and an adhesion promoter (TI Prime, MicroChemicals GmbH) was applied via spin-coating. Then, resin IP-S (Nanoscribe GmbH & Co. KG) was applied. The designs were made using the Nanoscribe GT and the Nanoscribe QuantumX align systems in a layer-by-layer fashion. A lateral hatching distance of 0.3 μm and a vertical slicing distance of 0.2 μm were used. To ensure good print quality, while using a 25× magnification objective, the structures were split into cubes with 250 μm edge length. After the structures were exposed, they were developed using propylene glycol methyl ether acetate, rinsed with isopropanol, and blow dried with nitrogen.

### Molding Process

From the DLW-made master molds, microscaffolds were prepared in a multistep casting process. First, master prints were silanized to lower the adhesion of the casting material. Vapor deposition of trichloro­(1H,1H,2H,2H-perfluorooctyl)­silane (PFOCTS; Sigma-Aldrich/Merck) was performed by placing samples on a tray over a few drops of PFOCTS in a desiccator. Pressure was reduced to −0.8 bar for 60 min, and samples were baked at 90 °C for 15 min subsequently. Base and curing agent of polydimethylsiloxan (PDMS; SYLGARD 184, Dow) was mixed thoroughly (10:1), centrifuged until completely degassed, and given on the master. After additional degassing in a vacuum chamber, PDMS was cured at room temperature (RT) for 24 h and subsequently silanized as described before. Epoxy resin (resin L + hardener L, R&G Faserverbundwerkstoffe GmbH) was prepared by mixing the base and hardener in the ratio of 100:40, degassing via centrifugation, and directly casting onto the PDMS positive replica. Curing for 24 h at RT was followed by separating PDMS and epoxy and fully curing epoxy at 60 °C for 5 h. After the epoxy negative replica was silanized, another PDMS positive replica was manufactured and silanized. To create molds for microfluidic fabrication, epoxy resin was given on the PDMS positive replica, gently degassed in a negative pressure chamber, pressed on a glass substrate, and taken off after curing at RT overnight. Excess material was removed, and the mold was hardened at 60 °C. A partially closed frame of 100 μm thick transparency film was glued around the central structures. The molds were again silanized before microscaffold manufacturing.

### Microscaffold Fabrication

PDMS was prepared as described previously, poured onto the glass-epoxy molds, and degassed in a vacuum chamber. A piece of transparency film was placed over the liquid PDMS in the frame and covered by a spacer made of PDMS and a glass layer. With a clamp, the sandwich was pressed and placed in an oven to cure at 60 °C for 30 min. After removing the microfluidics from the mold and frame, we verified visually the structural integrity and that all microwells were open from the top. If not, we tried to get rid of the thin PDMS layer covering them by gently scratching with a tweezer.

### Antifouling Surface Coating

To prevent neurons extending on top of the microfluidic devices, we functionalized them with an antifouling surface coating as described previously.^13,14^ Briefly, 13 mg of poly­(allylamine hydrochloride) (PAAm; Sigma-Aldrich/Merck) was dissolved with 31.8 mg of potassium carbonate (Sigma-Aldrich/Merck) in 2.6 mL of ultrapure water. The mixture was heated to boiling for improved dissolution and then allowed to cool rapidly. Separately, 11.2 mg of N-succinimidyl 4-azido-2,3,5,6-tetrafluorobenzoate (ATFB-NHS; Iris Biotech) was dissolved in 4.135 mL of pure ethanol (EtOH) and protected from light, briefly ultrasonicated (∼10 s), and gradually added to the PAAm solution under continuous stirring with a magnetic stirrer. Stirring was performed for at least 3 h to achieve a clear solution. If precipitation occurred, the mixture was discarded and the process repeated. For further steps, the PAAm-ATFB solution was diluted to 0.1 mg/mL in a 2:3 mixture of HEPES/EtOH [10 mM N-(2-hydroxyethyl)­piperazine-N′-(2-ethanesulfonic acid) (HEPES) in ultrapure water; Sigma-Aldrich/Merck]. To functionalize PDMS, all microscaffolds were surface-activated in a plasma device (ambient air, 0.3 mbar, 18 W power, 2 min; Diener electronic). The PAAm-ATFB solution was directly given on the microscaffolds to cover them completely and left to be protected from light at RT for 30 min. Then, PDMS was washed with HEPES/EtOH and ultrapure water. 10 mg/mL polyvinylpyrrolidone (Sigma-Aldrich/Merck) in EtOH was added on top of the PDMS, excess solution was taken off, and everything was blow dried with pressured air. The coating was then exposed to ultraviolet light in a cell culture hood for 5 min. To remove excess coating solutions, PDMS was rinsed in methanol for 1 h, with the methanol replaced every 15 min. Ultrasonication of microfluidics in fresh methanol for 5 min was done before washing with ultrapure water and storing of antifouling-coated microscaffolds in ultrapure water at 4 °C.

### Preparation of EMX1 Cell Line

EMX1 open reading frame was synthesized (Gen9, Inc.) and cloned into the doxycycline-inducible, puromycin-selectable lentiviral backbone pLIX_403 (Addgene #41395) using BP/LR Clonase (Thermo Fisher Scientific) following manufacturer protocols. Lentiviral particles were produced by cotransfecting pLIX_403+EMX1, pMD2G (Addgene #12259), and psPAX2 (Addgene #12260) into HEK293T cells using polyethylenimine (PolyScience, Inc.) as previously described.^64^ iNGN cells were transduced with pLIX_403+EMX1 lentiviral particles and then selected for 48 h post-transduction using 3 μg/mL puromycin. For live cell microscopy, EMX1 cells were genetically modified to constitutively overexpress tdTomato.

### Stem Cell Culture and Neuronal Differentiation

Human neurons were generated by the overexpression of neurogenic TFs in hiPSCs. iNGN neurons were prepared as previously described.^9,48,65^ By overexpression of TFs neurogenin-1 and neurogenin-2 either with or without EMX1 under a TetON inducible promoter system, hiPSCs differentiate within 4 days into postmitotic neurons (either iNGN or EMX1). For live cell microcopy, iNGN cells were genetically modified to constitutively overexpress EGFP. After thawing, uninduced stem cells were cultured on Matrigel (Corning)-coated plates with mTeSR1 medium (Stemcell Technologies), consisting of mTeSR1 basal medium with mTeSR1 supplement and 1% penicillin-streptomycin (P/S; Thermo Fisher Scientific) added. The medium was changed every day. Cultures were passaged before reaching confluency by adding TrypLE for 3 min and centrifuging at 359*g* for 4 min. After seeding, mTeSR1 was supplemented with the RHO/ROCK pathway inhibitor (ROCKi; Y-27632, Stemcell Technologies) for 24 h. Following at least two passages after thawing, differentiation of stem cells was induced on Matrigel-coated plates starting the day after passage by the addition of 0.5 μg/mL doxycycline (Dox; Sigma-Aldrich/Merck). Regular mycoplasma testing was performed on all cell cultures.

### Astrocyte Culture

Astrocytes were prepared by expanding rat primary astrocytes (A1261301, Thermo Fisher Scientific) in cell culture flasks and used up to P4. Astrocyte media consisted of DMEM with 4.5 g/L d-glucose, pyruvate, N2 supplement, 10% One Shot fetal bovine serum, and 1% P/S, all from Thermo Fisher Scientific. Astrocytes were cultured until they were almost confluent before reseeding in microscaffolds.

### Single-Cell Seeding

For the preparation of defined neuronal circuits with single-cell precision, a custom-built setup was used (Figure S1b). It consists of a cell culture microscope (Evos XL Core, Thermo Fisher Scientific) with a microinjector (IM-11–2, Narishige) mounted on a micromanipulator (MP-225, Sutter Instruments) under a sterile cell culture hood. MEA chips (60 electrode MEAs, 60MEA200/30iR-Ti, Multi Channel Systems) or coverslips were treated with plasma (ambient air, 0.3 mbar, 50 W; Diener electronic), incubated with poly-d-lysine (Merck) overnight at 37 °C, 100% relative humidity (RH), and washed thrice with deionized sterile water. Microscaffolds with antifouling coating were then placed on the MEA or coverslip with a drop of ultrapure water and aligned to the microelectrodes with a tweezer on a microscope under the cell culture hood. Substrates were dried inside the hood to maintain sterility. Additionally, they were placed in a vacuum chamber for at least 20 min to evaporate all water and increase the attachment of the microfluidics. A rectangular frame cut from PDMS (∼3 mm wide and high frame with a ∼1 × 1 cm opening in the center) was placed around the microfluidics. After prewetting the microscaffold with ultrapure water and applying a short pulse of negative pressure to fill the microfluidic channels, the water was taken off and laminin (0.05 mg/mL; Sigma-Aldrich/Merck) was added before incubating overnight at 37 °C, 100% RH.

Rat primary astrocytes were reseeded on the microscaffolds, usually the day before single-cell seeding. Astrocytes were detached with Accutase, centrifuged at 359*g* for 4 min, and resuspended in astrocyte media. 30,000 cells in 70 μL of media were given inside the frame on the microfluidics and incubated for 1 h before filling the MEA with astrocyte media and culturing until single-cell seeding.

Just before single-cell seeding, hiPSC-derived neurons at 5–6 dpi were detached with Accutase, centrifuged (359*g*, 4 min), and resuspended in 1 mL of complete BrainPhys medium. Complete BrainPhys consisted of BrainPhys Neuronal Medium with 1% P/S, 2% NeuroCult SM1 neuronal supplement (all from STEMCELL Technologies), 1% N2 supplement-A (Thermo Fisher Scientific), 20 ng/mL recombinant human BDNF, 20 ng/mL recombinant human GDNF (both from Peprotech), and 200 nM ascorbic acid (Sigma-Aldrich/Merck).

To investigate the best combination of supplements for increased survival of single neurons, we added either ROCKi or the CEPT cocktail (trans-ISRIB, 0.7 μM, Cayman Chemical; chroman 1, 50 nM, MedchemExpress; emricasan, 5 μM, Cayman Chemical; 0.1% polyamine supplement, Sigma-Aldrich), as reported by Chen et al.,^52^ to the culturing medium. Typically, cytarabine (AraC) is added to differentiating stem cell cultures after induction and just before reseeding to eliminate undifferentiated and still dividing cells. Since AraC has been reported to be neurotoxic and to cause DNA damage,^66^ we tested omitting it. By adding CEPT without AraC treatment, the survival rate increased to about 62.5% of cells 28 days after seeding (Figure S1c).

Single-cell seeding was performed after detachment and resuspension of neurons in supplemented (-AraC + CEPT for the best performance) BrainPhys medium. Astrocyte medium was aspirated from the samples and washed once with DPBS+/+ (Thermo Fisher Scientific) and supplemented BrainPhys was added inside the PDMS frame. A small amount of BrainPhys was added to the surrounding area outside the frame so that the two media reservoirs were not connected. A drop of resuspended neurons was added to the surrounding media, with the frame preventing cells from flowing onto the microscaffolds. Using the custom setup, single neurons were picked up with a glass pipet (VESbl-12–0–0–55, BioMedical Instruments) from the outside of the frame and then transferred to the inside and placed in a microwell. The correct placement of the cell in the microwell was visually confirmed before repeating the procedure until all microwells contained a neuron. The media outside the edge was aspirated, the area was gently washed, and the MEA was covered with a lid^67^ and placed in the incubator. After the cells had attached, the MEAs were filled with BrainPhys. Five days after the initial seeding of single neurons into the microwells, a second round of seeding was performed to maximize the number of complete circuits. The procedure was repeated, and any microwells where the previously placed neuron had been lost were reseeded. The old media was collected from the MEAs and enough media was left in the MEA for the seeding procedure (PDMS edge filled with media, small amount in the surrounding area). After the neurons were placed, the environment was aspirated and washed, and the MEAs were left in the incubator for attachment. Later, media (1:1 old media and fresh complete BrainPhys with CEPT) was added (Figure S1a).

As a control, populations of neurons were seeded into the microscaffolds. This was done in parallel with single-cell seeding. After aspiration of the astrocyte medium and washing of the MEA, 15,000 resuspended neurons in 70 μL of complete BrainPhys with CEPT were added within the PDMS frame. A second round of seeding was performed with 15,000 neurons in 12 μL of media to fill all microwells with at least five neurons, as visually confirmed under the microscope. After the MEAs were left in the incubator for attachment, they were filled with complete BrainPhys with CEPT.

For testing the optogenetic stimulation of single neurons, we seeded single cells of the fChRimson-iNGN cell line in microscaffolds. The fChRimson-iNGN cell line contained a double-floxed inverse open reading frame (DIO) of the fChRimson-EYFP gene.^60^ The fChRimson-EYFP sequence was kindly provided by Ernst Bamberg. The expression of fChRimson was induced by transfecting the cells with Cre recombinase mRNA; thereby, the fChRimson gene was in the frame. The DIO system can be used to delay the expression of the optogene until after the neuronal differentiation has been started by overexpression of TFs.^68^ This is crucial since stem cells often lose the expression of transgenes, especially optogenes. The fChRimson is a fast variant of the red-shifted channelrhodopsin ChRimson with an absorption maximum at around 590 nm wavelength of light.^60,69^ The mRNA was prepared by amplifying Cre DNA via PCR using the following primers: T7-Cre-forward: 5′-GCTAATACGACTCACTATAGGGACAGGCCACCATGGCCAATTTACTGA-3′ and T7-Cre-reverse: 5′-TCATTACGGTCCATCGCCATCTTCCAGCAGGCGCACCATT-3′ from the pCAG-Cre:GFP plasmid. pCAG-Cre:GFP was a gift from Connie Cepko (Addgene plasmid # 13776).^70^ The HiScribe T7 ARCA mRNA Kit (with tailing) (New England Biolabs) was used to create mRNA and was subsequently cleaned up with the Monarch Spin RNA Cleanup Kit (50 μg) (New England Biolabs). After transfecting the cells with Cre-mRNA using Lipofectamine MessengerMAX (Thermo Fisher Scientific) according to the manufacturers protocol at 0 dpi, the expression cassette in the cells’ gene was flipped and fChRimson was expressed. Cells were seeded as described above.

### Electrophysiology Experiments

Regular MEA recordings were performed on a MEA2100-Lite-System (Multi Channel Systems) with MC_Rack software (Multi Channel Systems). Imaging was done using a Keyence BZ-X810 with a 20× Keyence BZ-PF20LP objective. Recordings were performed for at least 3 min, usually 5–10 min at following dpi (±1 day) 13, 22, 27, 33, 39, 49 for Y-structures and dpi 14, 18, 21, 24, 27, 31, 33, 36, 49, 54, 61, 67, 76 for directional structures. Half media was exchanged weekly, and fresh complete BrainPhys medium was added.

Electrical stimulation was performed by using the MC_Rack software. After recording the baseline activity of neuronal circuits, a train of stimulation pulses (30 biphasic pulses consisting of −15 μA for 100 μs followed by 15 μA for 100 μs with an interpulse interval of 1800 ms) was applied to the left and right microchannels of the directional structures while recording the activity.

Optical stimulation of fChRimson-iNGN cells was done by placing the MEA headstage under an upright microscope (Scientifica SliceScope) with a 10× objective (Olympus Plan N 10×/0.25). A LED light source (pE-800, CoolLED) was connected to the microscope. A protocol for the light stimulation was prepared with Clampex 11.1. software that controlled the light source. Stimulation time stamps and MEA electrophysiology data were recorded with MC_Rack. The same stimulation protocol was applied with two different irradiances: 23 and 46 μW/mm^2^. Low irradiance levels were used to avoid a possible cytotoxic effect on single neurons and to prevent stimulation artifacts in the MEA electrodes. Stimulation was performed through the lid of the MEA to keep it sterile. Diameter of the light spot was 2 mm and was centered on the electrode area, covering it fully. The protocol consisted of trains of 50 ms light pulses with 0.5, 1, 2, and 5 Hz followed by 100 ms light pulses with 0.5, 1, and 2 Hz.

To block transmission between neurons through excitatory or electrical synapses, antagonists were added to the cultures. Excitatory NMDA and AMPA synapses were blocked by adding DL-2-amino-5-phosphonopentanoic acid (AP5; Tocris) and 2,3-dioxo-6-nitro-1,2,3,4-tetrahydrobenzo­[*f*]­quinoxaline-7-sulfonamide disodium salt (NBQX; Tocris), while electrical synapses (gap junctions) were blocked using CBX (Sigma-Aldrich/Merck). First, a baseline of activity was recorded for all samples. If stimulation was part of the overall experiment, a stimulation protocol was also applied under all conditions. Part of the medium was removed so that the cultures were covered still and collected for later use. NBQX (10 μM final concentration) and AP5 (50 μM final concentration) were added to the media and incubated for 30 min. Then, the activity was recorded again. After aspiration of the media and washing of samples (1× DPBS+/+, 2× fresh BrainPhys), the collected old media were added to the culture and incubated for 1 h before recording the activity again. CBX (100 μM final concentration) was added in the second step by using the same protocol.

### Data Analysis of Electrophysiological Recordings

Electrophysiology data were processed with custom-made python scripts using the SpikeInterface package.^71^ Preprocessing of data consisted of a second-order high-pass Butterworth filter with a cutoff at 100 Hz and a subsequent global common median referencing step.^72^ In SNAP recordings, spike sorting algorithms were not able to reliably separate spike trains of single neurons from the activity reliably. Multiple spike sorting algorithms that we tested consistently failed to provide accurate estimates of the number of neurons present in our circuits, instead drastically overestimating them (Figure S2e). This challenge is consistent with previous reports showing that isolating neuronal signals is particularly difficult in conditions of densely packed and synchronous APs, as observed in our recordings.^73,74^ Temporally overlapping waveforms and waveform superposition disrupt conventional spike sorting algorithms that rely on methods such as template matching and ultimately reduce the signal-to-noise ratio.

Therefore, we adapted our analysis strategy to a simpler electrodewise spike detection approach and omitted sorting for all analyzed data. We extracted all peaks in the traces of all electrodes in a circuit applying a standard algorithm implemented in the detect_peaks function of the SpikeInterface package, using the following criteria: negative signs only, detection threshold: 5 × median absolute deviations; exclude_sweep_ms: 0.2. Detected peaks and their timestamps were used as spike trains for further analysis of the network activity.

AP velocities were extracted from preprocessed raw traces by using a custom-made python script. Extracted peaks or timestamps are not sufficiently reliable for calculating AP velocities. During bursts, the same peak or AP potential cannot be identified unambiguously across several electrodes. This is because of temporally overlapping waveforms and waveform superposition as well as changes in AP waveform due to propagation or the position of the recording electrode.^74,75^ For this reason, we chose to use the initial rising phase of the voltage trace (whether from a single AP or a burst) as the reference point. Following a classical thresholding approach, we defined the reference time point as the moment when the voltage rose beyond five standard deviations of the baseline. This value was extracted for each electrode using the same algorithm. Because electrode spacing is known, this allowed us to calculate the propagation speed. To determine the standard deviation for the threshold, a time window for calculation had to be defined. We consistently used a 10 ms window for all analyses. Given the sparse firing in our samples (below 6 Hz on average, as shown in Figure 5b), this 10 ms window provides a sufficient estimate of the baseline and background noise while still retaining most of the traces. Spikes in the recording were chosen at random and an estimate of the time point in the range of 1 ms before the AP was fed into our custom algorithm. The algorithm then calculated the standard deviation of the signal during the 10 ms before and extracted the time point when the trace rose above the threshold of 5 times the calculated standard deviation. This was done in all electrodes, and time points of the threshold crossings were used to calculate the propagation velocities between the electrodes. Distances were known from the microfluidic designs. A minimum of 10 APs per condition, sample, and time point were extracted. If during the 10 ms before the AP there was another AP visible in the trace, the AP was rejected for measurement since the calculation of standard deviation would be distorted. Data acquisition and processing were performed identically across all circuits, conditions, and experiments.

To check for changes in firing regularity, burstiness, and synchronized bursting, which are signs of functional excitatory connections, we looked at ISI statistics. Due to the small circuit size and limited number of neurons and electrodes, signals from individual neurons were detected on multiple electrodes (see Figure 2d). Correlation analysis can thus not capture changes in firing among electrodes. Using ISI allows us to directly quantify changes in the regularity of activity patterns from single electrodes without the need for sorting single neuron data. ISI histograms were prepared by binning all time intervals between two consecutive spikes extracted in an electrode (1 ms bin size). The Kullback–Leibler divergence^55^ of two probability distributions *P*(*x*) and *Q*(*x*) is defined as

$$\mathrm{KL}(P,Q)=\sum_{x}P(x)\log\left(\frac{P(x)}{Q(x)}\right)$$

and gives a measure of how much two distributions differ. This measure was applied to ISI histograms, comparing histograms of baseline activity and activity under excitatory blockers. All electrodes in a motif with SNAP circuits or populations of neurons were evaluated separately. To avoid artifacts in the calculation of the KL divergence because of sparse histograms, we only included histograms with more than 400 ISI values in the analysis.

As a measure of synchronicity in a circuit we used the spike-contrast algorithm^58^ via its implementation in the Elephant python package.^76^ Burst extraction was performed with a MaxInterval method^77^ with the following parameters: minimum interburst interval: 200 ms, minimum burst duration: 1 ms, minimum number of spikes in burst: 3, and maximum ISI in a burst: 10 ms. For extraction of synchronized network bursts, we accepted only bursts that were present in all electrodes in a window of 5 ms. To extract the PR of a circuit motif, we calculated the matrix of correlation coefficients of all binned spike trains in a circuit (5 ms bins). The PR is defined by

$$\mathrm{PR}=\frac{1}{N}\frac{{\left(\sum_{i}\lambda_{i}^{a}\right)}^{2}}{\sum_{i}(\lambda_{i}^{a}{)}^{2}}$$

where λ_*i*_^*a*^ are the eigenvalues of the matrix of the activation of *N* units.^59^ This ratio of 0 ≤ PR ≤ 1 gives an intuition of the dimensionality of the activity in a network. If the neurons’ activity is more decoupled, the dimensionality and the PR are large, and if the neurons’ activity is more coupled and correlated, the dimensionality and PR are smaller, meaning more neurons participate in the common activity.

For the analysis of responses to optogenetic stimulation, we extracted the AP peaks in a window of 150 ms after the light on time point. To exclude artifacts, we accepted only peaks with an amplitude greater than 50 μV.

### Immunofluorescence

Neuron cultures were fixed with 4% paraformaldehyde solution (Thermo Fisher Scientific) for 15 min at RT. After washing with PBS, the specimen were permeabilized with 0.2% Triton X-100 (Sigma-Aldrich/Merck) in PBS for 10 min and then blocked with 5% donkey serum (Sigma-Aldrich/Merck) in PBS for 30 min at RT. Primary antibodies were added overnight at 4 °C (anti-GFP, 1:200, A10262, Thermo Fisher Scientific; anti-beta-III-tubulin linked to eFluor 570, 1:200, 41–4510–80, Invitrogen). Washing was done 3× with 0.1% Triton X-100 in PBS. Secondary antibody was added in the blocking solution for 2 h at RT (goat anti-chicken Alexa Fluor 488, 1:500, A11039, Thermo Fisher Scientific). After washing 3× with 0.1% Triton X-100 in PBS, Hoechst stain (H3570, Thermo Fisher Scientific) was added for 5 min at RT (1:2000 in PBS). After washing 3× with PBS, samples were mounted in ProLong Diamond Antifade Mountant (Thermo Fisher Scientific). Imaging was performed on an Echo Revolve microscope.

### Statistical Analysis

Statistical analysis was performed in GraphPad Prism version 10.4.1 for Windows, GraphPad Software, Boston, Massachusetts USA, www.graphpad.com. Plotting of data was done in GraphPad Prism and with custom-made python scripts. Statistical tests are mentioned in the figure captions where applicable with the corresponding number of values. Survival rates were extracted by evaluating the number of neurons visible in the fluorescence microscopy images per circuit.

## Supplementary Material





## Notes

Preparation and time development of single neuron circuits and survival and functional analysis of directional circuits (PDF)
Single-cell seeding process (MP4)

## References

[1] Schmieder F., Habibey R., Striebel J., Büttner L., Czarske J., Busskamp V. (2022). Tracking Connectivity Maps in Human
Stem Cell-Derived Neuronal Networks by Holographic Optogenetics. Life Sci. Alliance.

[2] Müller J., Ballini M., Livi P., Chen Y., Radivojevic M., Shadmani A., Viswam V., Jones I. L., Fiscella M., Diggelmann R., Stettler A., Frey U., Bakkum D. J., Hierlemann A. (2015). High-Resolution CMOS MEA Platform to Study Neurons
at Subcellular, Cellular, and Network Levels. Lab Chip.

[3] Barral J., Reyes A. D. (2016). Synaptic Scaling Rule Preserves Excitatory-Inhibitory
Balance and Salient Neuronal Network Dynamics. Nat. Neurosci..

[4] Yamamoto H., Moriya S., Ide K., Hayakawa T., Akima H., Sato S., Kubota S., Tanii T., Niwano M., Teller S., Soriano J., Hirano-Iwata A. (2018). Impact of
Modular Organization on Dynamical Richness in Cortical Networks. Sci. Adv..

[5] Albers J., Offenhäusser A. (2016). Signal Propagation
between Neuronal Populations Controlled
by Micropatterning. Front Bioeng Biotechnol.

[6] Kunze A., Tseng P., Godzich C., Murray C., Caputo A., Schweizer F. E., Di Carlo D. (2015). Engineering Cortical Neuron Polarity
with Nanomagnets on a Chip. ACS Nano.

[7] Duru J., Küchler J., Ihle S. J., Forró C., Bernardi A., Girardin S., Hengsteler J., Wheeler S., Vörös J., Ruff T. (2022). Engineered Biological
Neural Networks on High Density CMOS Microelectrode Arrays. Front Neurosci.

[8] Peyrin J. M., Deleglise B., Saias L., Vignes M., Gougis P., Magnifico S., Betuing S., Pietri M., Caboche J., Vanhoutte P., Viovy J. L., Brugg B. (2011). Axon Diodes
for the
Reconstruction of Oriented Neuronal Networks in Microfluidic Chambers. Lab Chip.

[9] Habibey R., Striebel J., Schmieder F., Czarske J., Busskamp V. (2022). Long-Term
Morphological and Functional Dynamics of Human Stem Cell-Derived Neuronal
Networks on High-Density Micro-Electrode Arrays. Front Neurosci.

[10] Braganza O., Beck H. (2018). The Circuit Motif as a Conceptual
Tool for Multilevel Neuroscience. Trends Neurosci.

[11] Sporns O., Kötter R. (2004). Motifs in Brain Networks. PLoS
Biol..

[12] Marder E., Bucher D. (2007). Understanding Circuit
Dynamics Using the Stomatogastric
Nervous System of Lobsters and Crabs. Annu.
Rev. Physiol..

[13] Girardin S., Clément B., Ihle S. J., Weaver S., Petr J. B., Mateus J. C., Duru J., Krubner M., Forró C., Ruff T., Fruh I., Müller M., Vörös J. (2022). Topologically Controlled Circuits of Human IPSC-Derived
Neurons for Electrophysiology Recordings. Lab
Chip.

[14] Weydert S., Girardin S., Cui X., Zürcher S., Peter T., Wirz R., Sterner O., Stauffer F., Aebersold M. J., Tanner S., Thompson-Steckel G., Forró C., Tosatti S., Vörös J. (2019). A Versatile
Protein and Cell Patterning Method Suitable for Long-Term Neural Cultures. Langmuir.

[15] Forró C., Thompson-Steckel G., Weaver S., Weydert S., Ihle S., Dermutz H., Aebersold M. J., Pilz R., Demkó L., Vörös J. (2018). Modular Microstructure Design to Build Neuronal Networks
of Defined Functional Connectivity. Biosens
Bioelectron.

[16] Habibey R., Striebel J., Meinert M., Latiftikhereshki R., Schmieder F., Nasiri R., Latifi S. (2024). Engineered Modular
Neuronal Networks-on-Chip Represent Structure-Function Relationship. Biosens Bioelectron.

[17] Habibey R., Jesús J., Arias E. R., Striebel J., Busskamp V. (2022). Microfluidics
for Neuronal Cell and Circuit Engineering. Chem.
Rev..

[18] Harberts J., Fendler C., Teuber J., Siegmund M., Silva A., Rieck N., Wolpert M., Zierold R., Blick R. H. (2020). Toward
Brain-on-a-Chip: Human Induced Pluripotent Stem Cell-Derived Guided
Neuronal Networks in Tailor-Made 3D Nanoprinted Microscaffolds. ACS Nano.

[19] Amos, G. ; Vasiliauskaitė, V. ; Duru, J. ; Saramago, M. L. A. ; Schmid, T. ; Suter, A. ; Torren, F. C. ; Küchler, J. ; Ruff, T. ; Vörös, J. ; Vulić, K. An Integrated In Vitro Platform and Biophysical Modeling Approach for Studying Synaptic Transmission in Isolated Neuronal Pairs. bioRxiv 2025 10.1101/2025.06.04.657933. PMC13092622 42016309

[20] Dörig P., Stiefel P., Behr P., Sarajlic E., Bijl D., Gabi M., Vörös J., Vorholt J. A., Zambelli T. (2010). Force-Controlled
Spatial Manipulation of Viable Mammalian Cells and Micro-Organisms
by Means of FluidFM Technology. Appl. Phys.
Lett..

[21] Connolly, S. ; Vulić, K. ; Zare-Eelanjegh, E. ; Simonett, M. ; Duru, J. ; Ruff, T. ; Clément, B. F. ; Vörös, J. Constructing Well-Defined Neural Networks of Multiple Cell Types by Picking and Placing of Neuronal Spheroids Using FluidFM. bioRxiv 2024 10.1101/2024.09.03.610979.

[22] Katz B., Schmitt O. H. (1940). Electric Interaction
between Two Adjacent Nerve Fibres. J. Physiol.

[23] Anastassiou C. A., Montgomery S. M., Barahona M., Buzsáki G., Koch C. (2010). The Effect of Spatially Inhomogeneous Extracellular Electric Fields
on Neurons. J. Neurosci..

[24] Wei N., Tolkacheva E. G. (2022). Mechanisms
of Arrhythmia Termination during Acute Myocardial
Ischemia: Role of Ephaptic Coupling and Complex Geometry of Border
Zone. PLoS One.

[25] Lin J., Keener J. P. (2013). Ephaptic Coupling
in Cardiac Myocytes. IEEE Trans Biomed Eng..

[26] Stacey R. G., Hilbert L., Quail T. (2015). Computational Study of Synchrony
in Fields and Microclusters of Ephaptically Coupled Neurons. J. Neurophysiol.

[27] Rabinovitch A., Rabinovitch R., Smolik E., Biton Y., Braunstein D. (2024). Ephaptic Conduction
in Tonic-Clonic Seizures. Front Neurol.

[28] Pinotsis D. A., Miller E. K. (2023). In Vivo Ephaptic
Coupling Allows Memory Network Formation. Cerebral
Cortex.

[29] Schmidt H., Hahn G., Deco G., Knösche T. R. (2021). Ephaptic
Coupling in White Matter Fibre Bundles Modulates Axonal Transmission
Delays. PLoS Comput. Biol..

[30] Capllonch-Juan M., Sepulveda F. (2020). Modelling
the Effects of Ephaptic Coupling on Selectivity
and Response Patterns during Artificial Stimulation of Peripheral
Nerves. PLoS Comput. Biol..

[31] Schmidt H., Knösche T. R. (2019). Action
Potential Propagation and Synchronisation in
Myelinated Axons. PLoS Comput. Biol..

[32] Anastassiou C. A., Perin R., Markram H., Koch C. (2011). Ephaptic Coupling
of
Cortical Neurons. Nat. Neurosci.

[33] Han K. S., Guo C., Chen C. H., Witter L., Osorno T., Regehr W. G. (2018). Ephaptic
Coupling Promotes Synchronous Firing of Cerebellar Purkinje Cells. Neuron.

[34] Blot A., Barbour B. (2014). Ultra-Rapid Axon-Axon Ephaptic Inhibition
of Cerebellar
Purkinje Cells by the Pinceau. Nat. Neurosci.

[35] Han K. S., Chen C. H., Khan M. M., Guo C., Regehr W. G. (2020). Climbing
Fiber Synapses Rapidly and Transiently Inhibit Neighboring Purkinje
Cells via Ephaptic Coupling. Nat. Neurosci.

[36] Bokil H., Laaris N., Blinder K., Ennis M., Keller A. (2001). Ephaptic Interactions
in the Mammalian Olfactory System. J. Neurosci..

[37] Vroman R., Klaassen L. J., Kamermans M. (2013). Ephap Tic
Communication in the Vertebrate
Retina. Front Hum Neurosci.

[38] Kamermans M., Fahrenfort I., Schultz K., Janssen-Bienhold U., Sjoerdsma T., Weiler R. (2001). Hemichannel-Mediated Inhibition in
the Outer Retina. Science.

[39] Weiss S. A., Faber D. S. (2010). Field Effects in
the CNS Play Functional Roles. Front Neural
Circuits.

[40] Lin J., Abraham A., George S. A., Greer-Short A., Blair G. A., Moreno A., Alber B. R., Kay M. W., Poelzing S. (2022). Ephaptic Coupling Is a Mechanism
of Conduction Reserve
During Reduced Gap Junction Coupling. Front
Physiol.

[41] Dudek F. E., Yasumura T., Rash J. E. (1998). ‘Non-Synaptic’ Mechanisms
in Seizures and Epileptogenesis. Cell Biol.
Int..

[42] Shivacharan R. S., Chiang C. C., Wei X., Subramanian M., Couturier N. H., Pakalapati N., Durand D. M. (2021). Neural Recruitment
by Ephaptic Coupling in Epilepsy. Epilepsia.

[43] Subramanian M., Chiang C. C., Couturier N. H., Durand D. M. (2022). Theta Waves, Neural
Spikes and Seizures Can Propagate by Ephaptic Coupling in Vivo. Exp. Neurol..

[44] Hoagland D. T., Santos W., Poelzing S., Gourdie R. G. (2019). The Role of the
Gap Junction Perinexus in Cardiac Conduction: Potential as a Novel
Anti-Arrhythmic Drug Target. Prog. Biophys.
Mol. Biol..

[45] Veeraraghavan R., Hoeker G. S., Laviada A. A., Hoagland D., Wan X., King D. R., Alonso J. S., Chen C., Jourdan J., Isom L. L., Deschenes I., Smith J. W., Gorelik J., Poelzing S., Gourdie R. G. (2018). The Adhesion Function of the Sodium
Channel Beta Subunit (Β1) Contributes to Cardiac Action Potential
Propagation. Elife.

[46] Dworak B. J., Wheeler B. C. (2009). Novel MEA Platform with PDMS Microtunnels Enables the
Detection of Action Potential Propagation from Isolated Axons in Culture. Lab Chip.

[47] Taylor A. M., Blurton-Jones M., Rhee S. W., Cribbs D. H., Cotman C. W., Jeon N. L. (2005). A Microfluidic Culture Platform for CNS Axonal Injury. Regeneration and Transport. Nat. Methods.

[48] Busskamp V., Lewis N. E., Guye P., Ng A. H., Shipman S. L., Byrne S. M., Sanjana N. E., Murn J., Li Y., Li S., Stadler M., Weiss R., Church G. M. (2014). Rapid Neurogenesis
through Transcriptional Activation in Human Stem Cells. Mol. Syst. Biol..

[49] Wang Z. Q., Kiefer F., Urbánek P., Wagner E. F. (1997). Generation of Completely
Embryonic Stem Cell-Derived Mutant Mice Using Tetraploid Blastocyst
Injection. Mech. Dev..

[50] Gossler A., Doetschman T., Korn R., Serfling E., Kemler R. (1986). Transgenesis
by Means of Blastocyst-Derived Embryonic Stem Cell Lines. Proc. Natl. Acad. Sci. U. S. A..

[51] Watanabe K., Ueno M., Kamiya D., Nishiyama A., Matsumura M., Wataya T., Takahashi J. B., Nishikawa S., Nishikawa S. I., Muguruma K., Sasai Y. (2007). A ROCK Inhibitor
Permits Survival of Dissociated Human Embryonic Stem Cells. Nat. Biotechnol..

[52] Chen Y., Tristan C. A., Chen L., Jovanovic V. M., Malley C., Chu P. H., Ryu S., Deng T., Ormanoglu P., Tao D., Fang Y., Slamecka J., Hong H., LeClair C. A., Michael S., Austin C. P., Simeonov A., Singeç I. (2021). A Versatile
Polypharmacology Platform
Promotes Cytoprotection and Viability of Human Pluripotent and Differentiated
Cells. Nat. Methods.

[53] Lam R. S., Töpfer F. M., Wood P. G., Busskamp V., Bamberg E. (2017). Functional
Maturation of Human Stem Cell-Derived Neurons in Long-Term Cultures. PLoS One.

[54] Kunze, S. Created in BioRender, 2025. Https://BioRender.Com/Rxqdqiu.

[55] Kullback S., Leibler R. A. (1951). On Information and
Sufficiency. Annals of Mathematical Statistics.

[56] Miyamoto K. I., Ichimura H., Wagner T., Schöning M. J., Yoshinobu T. (2013). Chemical Imaging of the Concentration Profile of Ion
Diffusion in a Microfluidic Channel. Sens Actuators
B Chem..

[57] Habibey R., Latifi S., Mousavi H., Pesce M., Arab-Tehrany E., Blau A. (2017). A Multielectrode Array Microchannel Platform Reveals Both Transient
and Slow Changes in Axonal Conduction Velocity. Sci. Rep.

[58] Ciba M., Isomura T., Jimbo Y., Bahmer A., Thielemann C. (2018). Spike-Contrast:
A Novel Time Scale Independent and Multivariate Measure of Spike Train
Synchrony. J. Neurosci Methods.

[59] Clark D. G., Abbott L. F., Litwin-Kumar A. (2023). Dimension
of Activity in Random Neural
Networks. Phys. Rev. Lett..

[60] Mager T., Morena D. L. D. La, Senn V., Schlotte J., Derrico A., Feldbauer K., Wrobel C., Jung S., Bodensiek K., Rankovic V., Browne L., Huet A., Jüttner J., Wood P. G., Letzkus J. J., Moser T., Bamberg E. (2018). High Frequency
Neural Spiking and Auditory Signaling by Ultrafast Red-Shifted Optogenetics. Nat. Commun..

[61] Permana S., Grant E., Walker G. M., Yoder J. A. (2016). A Review of Automated
Microinjection Systems for Single Cells in the Embryogenesis Stage. IEEE/ASME Transactions on Mechatronics.

[62] Gross A., Schöndube J., Niekrawitz S., Streule W., Riegger L., Zengerle R., Koltay P. (2013). Single-Cell Printer: Automated, On
Demand, and Label Free. J. Lab Autom.

[63] Ming Y., Abedin M. J., Tatic-Lucic S., Berdichevsky Y. (2021). Microdevice
for Directional Axodendritic Connectivity between Micro 3D Neuronal
Cultures. Microsyst Nanoeng.

[64] Ng A. H. M., Khoshakhlagh P., Rojo Arias J. E., Pasquini G., Wang K., Swiersy A., Shipman S. L., Appleton E., Kiaee K., Kohman R. E., Vernet A., Dysart M., Leeper K., Saylor W., Huang J. Y., Graveline A., Taipale J., Hill D. E., Vidal M., Melero-Martin J. M., Busskamp V., Church G. M. (2021). A Comprehensive
Library of Human
Transcription Factors for Cell Fate Engineering. Nat. Biotechnol..

[65] Striebel J., Kalinski L., Sturm M., Drouvé N., Peters S., Lichterfeld Y., Habibey R., Hauslage J., El Sheikh S., Busskamp V., Liemersdorf C. (2023). Human Neural
Network Activity Reacts to Gravity Changes in Vitro. Front Neurosci.

[66] Geller H. M., Cheng K. Y., Goldsmith N. K., Romero A. A., Zhang A. L., Morris E. J., Grandison L. (2001). Oxidative
Stress Mediates Neuronal
DNA Damage and Apoptosis in Response to Cytosine Arabinoside. J. Neurochem.

[67] Blau A., Neumann T., Ziegler C., Benfenati F. (2009). Replica-Moulded
Polydimethylsiloxane Culture Vessel Lids Attenuate Osmotic Drift in
Long-Term Cell Cultures. J. Biosci.

[68] Klapper S. D., Sauter E. J., Swiersy A., Hyman M. A. E., Bamann C., Bamberg E., Busskamp V. (2017). On-Demand
Optogenetic Activation
of Human Stem-Cell-Derived Neurons. Sci. Rep.

[69] Klapoetke N. C., Murata Y., Kim S. S., Pulver S. R., Birdsey-Benson A., Cho Y. K., Morimoto T. K., Chuong A. S., Carpenter E. J., Tian Z., Wang J., Xie Y., Yan Z., Zhang Y., Chow B. Y., Surek B., Melkonian M., Jayaraman V., Constantine-Paton M., Wong G. K. S., Boyden E. S. (2014). Independent
Optical Excitation of Distinct Neural Populations. Nat. Methods.

[70] Matsuda T., Cepko C. L. (2007). Controlled Expression of Transgenes Introduced by in
Vivo Electroporation. Proc. Natl. Acad. Sci.
U. S. A..

[71] Buccino A. P., Hurwitz C. L., Garcia S., Magland J., Siegle J. H., Hurwitz R., Hennig M. H. (2020). Spikeinterface, a Unified Framework
for Spike Sorting. Elife.

[72] Rolston J. D., Gross R. E., Potter S. M. (2009). Common
Median Referencing for Improved
Action Potential Detection with Multielectrode Arrays. Annu. Int. Conf. IEEE Eng. Med. Biol. Soc..

[73] Garcia S., Buccino A. P., Yger P. (2022). How Do Spike Collisions
Affect Spike
Sorting Performance?. eNeuro.

[74] Bakkum D. J., Frey U., Radivojevic M., Russell T. L., Müller J., Fiscella M., Takahashi H., Hierlemann A. (2013). Tracking Axonal
Action Potential Propagation on a High-Density Microelectrode Array
across Hundreds of Sites. Nat. Commun..

[75] Gold C., Henze D. A., Koch C., Buzsáki G. (2006). On the Origin
of the Extracellular Action Potential Waveform: A Modeling Study. J. Neurophysiol.

[76] Denker, M. ; Yegenoglu, A. ; Grün, S. Collaborative HPC-Enabled Workflows on the HBP Collaboratory Using the Elephant Framework. In Neuroinformatics 2018; 2018; 19.

[77] Cotterill E., Charlesworth P., Thomas C. W., Paulsen O., Eglen S. J. (2016). A Comparison
of Computational Methods for Detecting Bursts in Neuronal Spike Trains
and Their Application to Human Stem Cell-Derived Neuronal Networks. J. Neurophysiol.

