# Supplementary material for: Reproducible Human Neural Circuits Printed with Single-Cell Precision Reveal the Functional Roles of Ephaptic Coupling
Source: ACS Nano. 2025 Oct 26;19(44):38457–71. doi: 10.1021/acsnano.5c11482 (PMC12613839; doi:10.1021/acsnano.5c11482)
Supplement: Supplementary file 1 [file nn5c11482_si_001.pdf]

Supporting Information

Reproducible Human Neural Circuits Printed with Single-Cell Precision Reveal the Functional Roles of Ephaptic Coupling

Johannes Striebel[Δ,±], Rouhollah Habibey[Δ,±], Daniel Wendland[Ⴈ,=], Helge Gehring[Ⴈ], Elizaveta Podoliak[Δ,±], Julia S. Pawlick[Δ,±], Kritika Sharma[Δ,±], Alex H. M. Ng[∨], Wolfram Pernice[Ⴈ,=], and Volker Busskamp[Δ,±,]*

[Δ] Faculty of Medicine, Department of Ophthalmology, University of Bonn, 53127 Bonn, Germany

[±] University Hospital Bonn, 53127 Bonn, Germany

[Ⴈ] Institute of Physics and Center for Nanotechnology, University of Münster, 48149 Münster, Germany

[=] Kirchhoff-Institute for Physics, University of Heidelberg, 69120 Heidelberg, Germany

[∨] Department of Genetics, Blavatnik Institute, Harvard Medical School, Boston, Massachusetts 02115, United States; Wyss Institute for Biologically Inspired Engineering at Harvard University, Boston, Massachusetts 02215, United States

[*] corresponding author: volker.busskamp@ukbonn.de

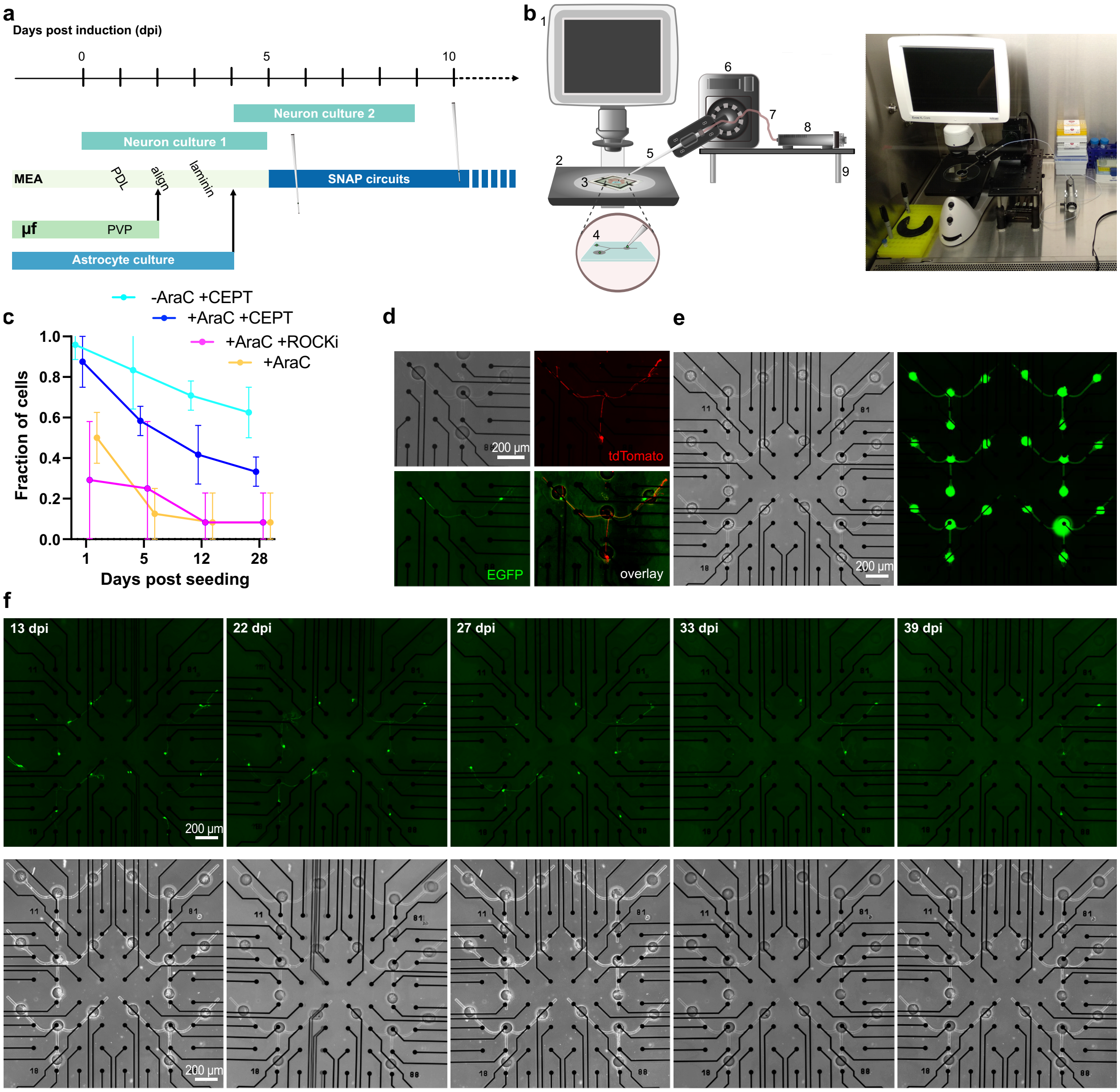

Figure S1. Preparation and time development of single neuron circuits. (a) Overview of the SNAP circuit preparation protocol. Single cell seedings are marked with a glass pipette icon. µf: microfluidics, MEA: multielectrode array, PVP: polyvinylpyrrolidone functionalization, PDL: Poly-D-Lysin coating. (b) Cell placement setup installed in a sterile cell culture hood and

consisting of a microscope (1) and stage (2) on which a MEA with a microscaffold (3) is placed for seeding (4). A micropipette (5) is steered by a micromanipulator (6). A silicon tube (7) connects the manual microinjector (8) to the pipette. All cell placement equipment is mounted on a custom-build and adjustable platform (9). An image of the physical setup under a sterile hood is shown on the right. (c) Comparison of cell survival under different preparation conditions. Fraction of surviving iNGN neurons is shown for different days post seeding. Adding CEPT cocktail and no AraC showed the best cell survival. N = 3 per condition and timepoint (mean ± SD). (d) Representative neuronal circuit motif consisting of two cell types: two iNGN (upper microwells) and one EMX1 (lower microwell) neurons connected through microchannels within a microfluidic device. Fluorescence images of tdTomato constitutively expressed by EMX1 neurons and constitutively expressed EGFP by iNGNs. (e) Representative MEA with populations of iNGN neurons seeded in microfluidics at 22 dpi (EGFP and brightfield channels). (f) Development of four Y-shaped motifs with three iNGNs each on one MEA is shown across time (EGFP and brightfield channels).

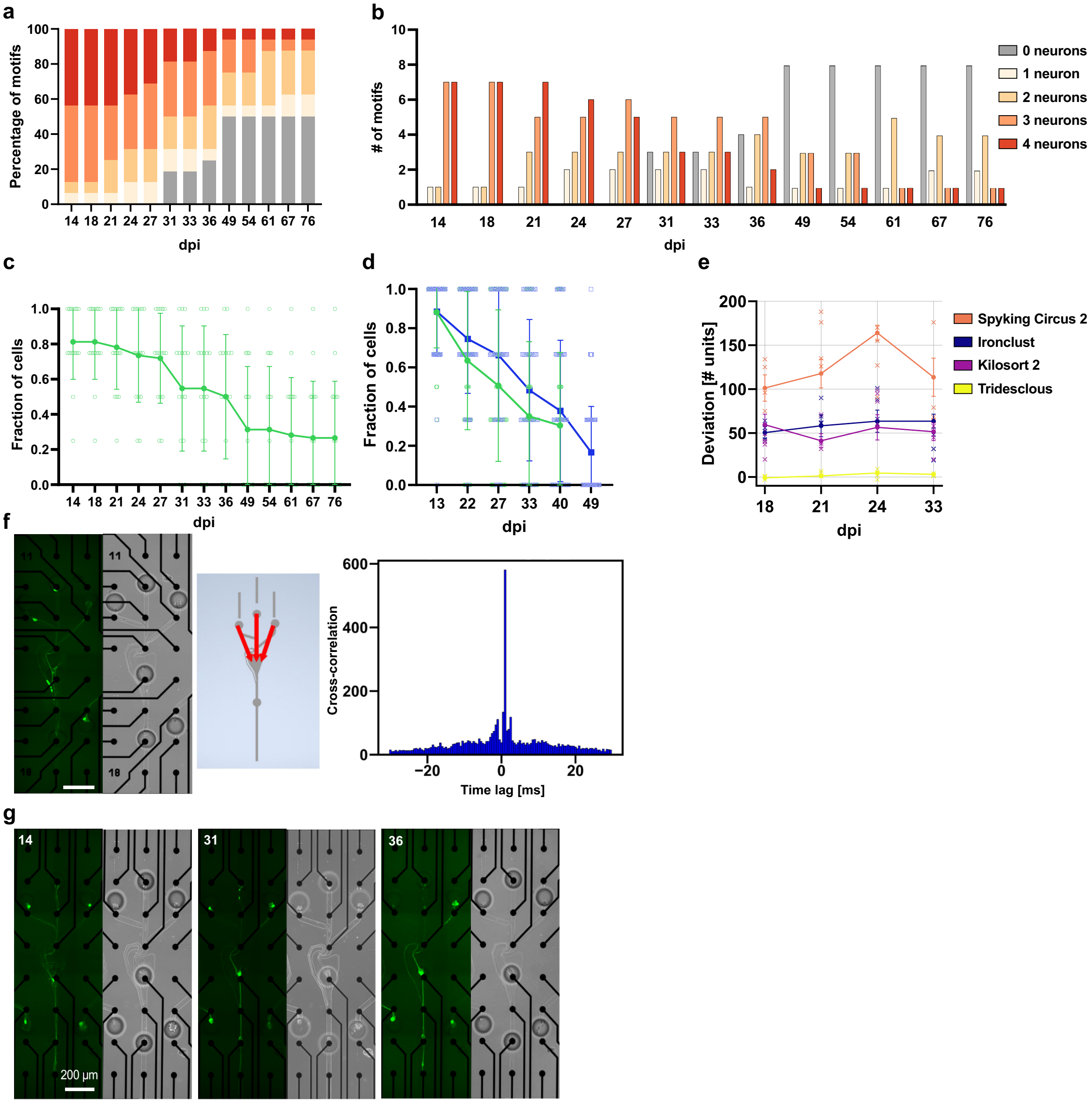

Figure S2. Survival and functional analysis of directional circuits. (a) Percentage of motifs containing the respective number of viable cells at several days post induction (dpi) for directional 4-neuron circuit motifs (iNGN: n = 16 circuits). (b) Number of circuit motifs containing respective number of iNGN neurons per dpi. 4 samples with 4 motifs each were monitored. (c) Fraction of viable cells at several dpi for directional 4-iNGN circuit motifs

(mean ± SD, iNGN: n = 16 circuits). Individual values are shown as empty circles. (d) Fraction of surviving iNGN (green) and EMX1 (blue) neurons in 3-neuron circuit motifs over time at different dpi (mean ± SD; iNGN: n = 64 circuits; EMX1: n = 60 circuits). Individual values are shown as empty circles or squares, respectively. (e) Performance of different spike sorting algorithms on directional circuits. Four algorithms were compared by extracting the number of units from four MEAs on four different dpi. The true number of units as observed in microscopy images was subtracted from the number of units found by the algorithms to get a deviation value. mean±s.e.m. (f) Representative analysis of signal propagation in a directional circuit of 4 iNGN neurons. Left: Fluorescence (constitutively expressed EGFP) and brightfield images of the circuit. Middle: Schematic of the microfluidic design overlayed with arrows indicating the direction of AP propagation as extracted by cross-correlation analysis of the signals measured in the electrodes of the respective microfluidic channels. Right: Representative cross-correlation analysis of signals in two electrodes measured in the circuit. (g) Fluorescence (constitutively expressed EGFP) and brightfield images of the circuit described in Fig. 4e with respective dpi mentioned.

Movie S1. Single cell seeding process. Cells are picked up by a glass micropipette from an area where a cell pool is kept and transferred to the electrode area of the multielectrode array with the microscaffold on top. A micromanipulator allows precise control of the micropipette in all three directions. A microinjector is used to pick up and release the cells. Speed of the video was tripled.
